# Systematic review of traditional Chinese medicine in treatment of dizziness

**DOI:** 10.1016/j.chmed.2026.08.008

**Published:** 2026-08-22

**Authors:** Charlotte McMahon, Chris Zaslawski, Vanessa Vigar, Nicole Hannan, Sophia Gerontakos, Janet Schloss

**Affiliations:** a National Centre for Naturopathic Medicine, Faculty of Health, Southern Cross University, Lismore 2480, Australia; b The University of Technology, Sydney 2007, Australia

**Keywords:** Chinese herbal medicine, dizziness, randomised controlled trials, traditional Chinese medicine, vertigo

## Abstract

Dizziness is a chronic condition that can significantly impact an individual’s quality of life (QoL) and increase risk of falls. It is characterised by a sense of environmental motion and loss of balance. It has been proposed that Chinese herbal medicine (CHM) can be used to effectively treat dizziness. This systematic literature review aims to evaluate the effectiveness of CHM on symptoms of dizziness and QoL. PubMed, Cumulative Index of Nursing and Allied Health Literature (CINAHL), Cochrane Central Register of Controlled Trials (CENTRAL), Medical Literature Analysis and Retrieval System Online (MEDLINE), China National Knowledge Infrastructure (CNKI) and Allied and Complementary Medicine Database (AMED) databases were searched between January 2010 to April 2025. Included studies were RCTs that assessed CHM for the treatment of dizziness. Non-RCTs, animal studies, *in vitro* studies, case reports and review articles were excluded. Primary outcome measures included overall treatment effectiveness, dizziness handicap inventory (DHI), total number of vertigo attacks, total number of days with vertigo attacks and severity of symptoms. A total of ten RCTs were included in this review, all studies used Chinese medicine diagnostic criteria and approached the treatment of dizziness from a traditional Chinese medicine (TCM) aetiology. Control interventions differed between studies with some using a placebo while others used experimental and/or positive controls. Duration of treatment ranged from 10 days to 16 weeks. All studies individually found CHM to be clinically effective in the treatment of dizziness (60%–90%) with improvements in symptom severity and QoL. No *meta*-analysis was conducted due to the high heterogeneity among studies. Safety and tolerability of CHM was high, however adverse event reporting was absent or minimal, limiting the depth of safety analysis. CHM has the potential to be safe and effective long-term treatment option in the management of patients experiencing acute and chronic presentation of dizziness.

## 1 Introduction

Dizziness is a broad term for a subjective experience and can be classified more precisely depending on predominant symptoms (Bösner et al., 2018). Vertigo is often described as a sense of environmental motion, disequilibrium presents as a feeling of loss of balance, presyncope defines the feeling of “about to lose consciousness”, whether or not the person has or has not lost consciousnesses, and light-headedness represents a floating or giddy feeling often isolated to the head (Bösner et al., 2018). Traditional Chinese medicine (TCM) is an ancient system of medicine that was the predominant form of healthcare in China until the 16th century (Dong, 2013). From the 16th century onward “Western medicine” was introduced and since then the medical system in China has developed into the integrated Chinese and Western medicine approach that is evident today at every level of the healthcare system in China (Dong, 2013, Hesketh and Zhu, 1997, Zhou et al., 2020, Yagüe et al., 2022). China is currently the predominant country utilising the combination of traditional and modern medicine in the mainstream treatment of patients, with the Chinese government including TCM development as a national strategy to achieve the Healthy China 2030 plan (Yagüe, Sun, & Hu, 2022). Other Asian countries such as Korea, and Japan also use integrated Chinese and Western medicine to varying degrees of inclusion.

Successfully achieving integrated Chinese and Western medicine is not without its challenges, as discussed in this literature review, Western medicine and TCM have very different perspectives on physiology and aetiology of disease and treatment. This can complicate the integration of these differing medical theories into effective clinical practice (Dong, 2013, Hesketh and Zhu, 1997). Western herbal medicine (WHM) has been developed from many traditional medicine systems including TCM and utilises many TCM herbal preparations while applying a Western medicine biomedical approach to disease aetiology. In this way WHM may be the bridge between the two systems, helping the Western medicine practitioner understand actions of herbs utilised in TCM from a Western medicine perspective.

Dizziness and vertigo generally affect around 15% to 20% of adults with vestibular vertigo accounting for a quarter of the dizziness complaints (Neuhauser, 2016). In Australia, the prevalence is even higher with 36.2% found to experience dizziness and vertigo (Gopinath, McMahon, Rochtchina, & Mitchell, 2009). Research into the effect of dizziness found that quality of life (QoL) was dramatically affected (Gopinath, McMahon, Rochtchina, & Mitchell, 2009). The functional burden of disease on patients who have vertigo, dizziness, and balance problems (VDB) may depend on the personality traits of the affected patients. What has been found is that patients with high self-efficacy and willingness to take risks may exhibit better coping mechanisms if faced with challenges compared to those with lower self-efficacy and willingness (Katzenberger et al., 2023). One large population study in an older cohort (average age 60.7 years) reports a vertigo prevalence of 21.6% in this group, finding that the symptoms were significantly bothersome for 8.1% of the population assessed, with females more bothered by their vertigo compared to males (Hackenberg et al., 2023). Moreover, it was shown that vertigo contributes significantly to the overall burden of disease (Hackenberg et al., 2023).

From a medical perspective, dizziness can be caused by a broad range of diseases from benign and self-limiting to severe and life-threatening. Western medicine pathophysiology determines the aetiologies of dizziness to be due to specific diseases such as vestibular neuritis/migraine, benign paroxysmal positional vertigo (BPPV) or Meniere’s disease or due to abnormalities in cardio-vascular, neurological, vestibular, ontological or psychogenic function (Bösner et al., 2018). The specific aetiology of dizziness may be determined based on laboratory testing and the type of dizziness experienced, vertigo may indicate dysfunction of the vestibular system, disequilibrium may indicate underlying disease in the neurological system and light-headedness and presyncope can have multiple causes, and are often difficult to diagnose with up to 80% of cases of dizziness having an unknown aetiology from a medical perspective (Bösner et al. 2018).

Due to dizziness and vertigo having various aetiologies, the treatment of the patient will vary based on their history and physical exam. It is crucial to accurately diagnose the cause to formulate the correct treatment plan (Pfieffer, Anthamatten, & Glassford, 2019). The most common cause is BPPV and the treatment for this is moving the patient into different positions and observing their eye movements (Cole & Honaker, 2022). However, due to the difficulty with diagnosis of dizziness and differential diagnoses due to the broad peripheral and central causes of the condition, treatment options can be limited, leading to reduced patient outcomes. Manual manipulation is the main treatment with pharmacological interventions being limited since they affect the ability of the central nervous system to compensate for dizziness (Rogers, Noel, & Garcia, 2023).

TCM views diseases from a holism perspective. The focus is on utilising herbal formulas to correct imbalances in the body. TCM utilises the theories of “syndrome differentiation and treatment” which considers the five *zang*-organs of liver, heart, spleen, lung and kidney and the six *fu*-organs of gallbladder, small intestine, stomach, large intestine, bladder and the triple energizer that can be in a state of excess or deficiency or produce pathological substances in the body such as phlegm or dampness. Furthermore, TCM postulates that the body has a system of twelve channels that circulate *qi* and blood between the organs of the body. These channels allow the organs to communicate in a way which supports whole body function and when these channels are blocked or become deficient in *qi* flow then disease arises (Ma et al., 2021). From this perspective, dizziness as a symptom can have many different aetiologies, therefore treatment approaches are different depending on the underlying cause.

TCM broadly classifies the pathogenesis of vertigo into four aspects: *Wind*, *fire*, phlegm, and deficiency (Zhang et al., 2021). Chinese herbal medicine (CHM) may be beneficial in improving symptoms of dizziness through dispelling *wind*, clearing away *fire*, resolving phlegm and resolving *qi* and blood (Zhang et al., 2021).

This literature review seeks to understand if CHM, either in isolation or combined with other treatment, may be beneficial in relieving dizziness, regardless of aetiology.

## 2 Methods

### 2.1 Inclusion and exclusion criteria

#### 2.1.1 Inclusion criteria

(1) Primary outcome

To analyse randomised control trials investigating the effectiveness of Chinese Herbal Medicine in the treatment of broad cause dizziness.

(2) Secondary outcomes

To determine which patient reported outcome measure were used in randomised clinical trials assessing TCM herbs for dizziness.

To assess the difference in treatment of dizziness from a TCM perspective compared to Western medicine. Different participant inclusion criteria were included across the studies. For details of the individual article inclusion criteria, see Table S1.

#### 2.1.2 Exclusion criteria

(1) Types of studies

A global search was conducted for randomised controlled trials (RCTs) written in English or Chinese that assessed CHM treatment for dizziness, regardless of the control intervention. Non-RCTs, animal or *in vitro* studies, case reports or review articles, and duplicate publications were excluded.

(2) Types of participants

Participants in the studies were those who experienced dizziness, vertigo, BPPV or had a diagnosis of chronic cerebral circulation insufficiency (CCCI). Participants were between 18–75 years of age, with no restriction on gender, ethnicity, duration or severity of disease.

(3) Types of interventions

RCTs investigating CHM for dizziness were included. Literature that did not include traditional CHM, or that utilised CHM for conditions other than dizziness, vertigo, CCCI or BPPV were excluded.

(4) Types of outcome measures

RCTs with clinical efficacy as the primary endpoint or main outcome were included. Primary outcome measures included dizziness handicap inventory (DHI) (Treleaven, 2006), total number of vertigo attacks, total number of days with vertigo attacks (Bayer, Adrion, Al Tawil, Mansmann, & Strupp, 2019), severity of symptoms (headache, heavy-headed feeling and sleep disorder), blood deficiency symptoms (BDS) grading and quantifying scale (Zhang et al., 2021), diagnostic and therapeutic efficacy criteria of TCM diseases (Wang et al., 2023), TCM syndrome scale (TCMSS) (Gan et al., 2023), clinical efficacy standards (cure/effective/ineffective), clinical efficacy standards according to TCMSS and clinical diagnosis and therapeutic effect criteria (Ding et al., 2025).

Secondary outcome measures included: QoL [typically through Short Form 36 score (SF-36)] (Kalland Knapstad, Goplen, Skouen, Ask, & Nordahl, 2020), self-rating anxiety scale (SAS) (Zung, 1971), self-rating depression scale (SDS) (Zung, 1965), autonomic symptom profile (ASP) (Low, Benarroch, Foster, & Vernino, 1997), blood flow velocity and other haematological indices.

### 2.2 Search strategy

This systematic literature review was registered with the International Prospective Register of Systematic Reviews (PROSPERO) (CRD42023390586). PubMed, Cumulative Index of Nursing and Allied Health Literature (CINAHL), Cochrane Central Register of Controlled Trials (CENTRAL), Medical Literature Analysis and Retrieval System Online (MEDLINE), China National Knowledge Infrastructure (CNKI) and Allied and Complementary Medicine Database (AMED) databases, which were searched between January 2010 to April 2025 to ensure that the latest research was being assessed. The search terms used included “dizzy” OR “vertigo” OR “dizziness” OR “orthostasis” OR “light-headedness” OR “spinning” AND “traditional Chinese medical practice and medicine” OR “traditional Chinese medical practice” OR “traditional Chinese medicine” OR “Chinese Herbal Medicine” OR “TCM” AND “Randomised controlled trial” OR “RCT” OR “Randomised Clinical Trial”. Studies written in either English or Chinese were included.

### 2.3 Data extraction

All included studies were written in Chinese and were translated into English using artificial intelligence (AI), in the form of google translate to enable data extraction. The translated articles were then reviewed by Chinese national academic, Dr. Wenbo Peng from Australian Research Centre in Complementary and Integrative Medicine (ARICCM) at University Technology of Sydney (UTS) and Associate Professor Chris Zaslawski from UTS to ensure accurate translation. Data was extracted by Nicole Hannan, Sophia Gerontakos, Vanessa Vigar, Janet Schloss and Charlotte McMahon and included information on study design, population description, participant numbers, age, gender, CHM used, outcome measures assessed and safety and tolerability data.

### 2.4 Critical appraisal

RCTs were evaluated using the Joanna Briggs Institute (JBI) critical appraisal checklist (Joanna Briggs Institute, 2017) to appraise the methodological qualities of the studies.

## 3 Results

### 3.1 Selection of studies

A total of 326 citations were identified of which 24 duplicates were removed resulting in 302 studies which were screened for title and abstract. After title and abstract screening, 47 papers were assessed for eligibility. Of these, 37 studies were excluded during full text screening due to: Wrong outcomes (3), wrong intervention (6), wrong study design (2), conference abstract (2), clinical trial register (9), wrong patient population (14), and wrong route of administration (1). A total of ten RCTs were included in this review (Fig. 1).

**Fig. 1:**
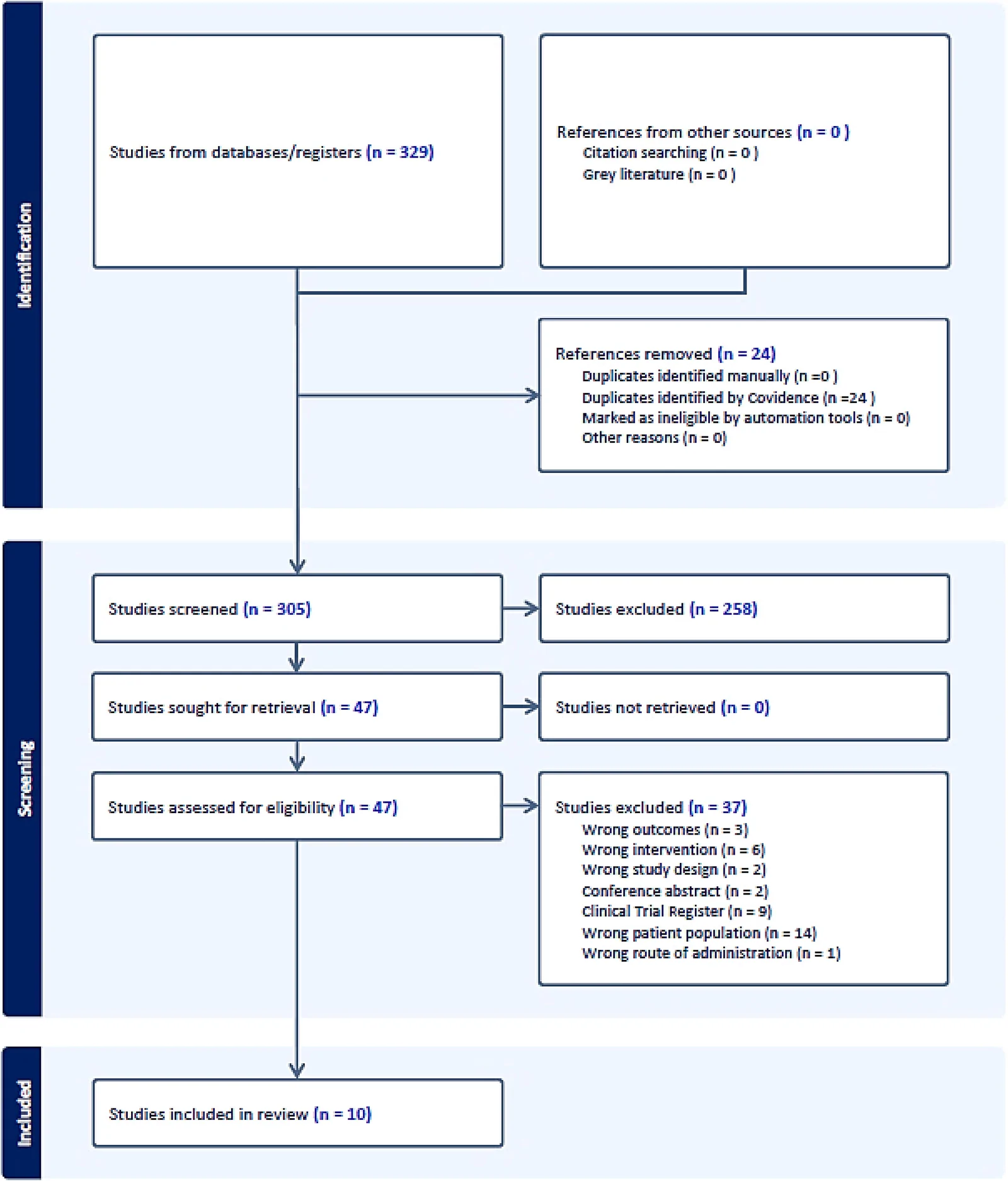
Prisma diagram.

### 3.2 Assessment of risk of bias

JBI critical appraisal tool for RCTs: (1) Was true randomization used for assignment of participants to treatment groups? (2) Was allocation to treatment groups concealed? (3) Were treatment groups similar at the baseline? (4) Were participants blind to treatment assignment? (5) Were those delivering treatment blind to treatment assignment? (6) Were outcomes assessors blind to treatment assignment? (7) Were treatment groups treated identically other than the intervention of interest? (8) Was follow up complete and if not, were differences between groups in terms of their follow up adequately described and analyzed? (9) Were participants analyzed in the groups to which they were randomized? (10) Were outcomes measured in the same way for treatment groups? (11) Were outcomes measured in a reliable way? (12) Was appropriate statistical analysis used? (13) Was the trial design appropriate, and any deviations from the standard RCT design (individual randomization, parallel groups) accounted for in the conduct and analysis of the trial? (14) Were RCTs included in literature review?

The lowest score on the JBI was seven while two of the articles scored (13) All studies included in this review were found to be at low risk of bias (Table 1).

**Table 1:** Critical appraisal of included studies.

Studies	Appraisal checklist criteria													
	1	2	3	4	5	6	7	8	9	10	11	12	13	14
Hao, Wang, Xie, Wang, & Zhang, 2024	Y	N	Y	N	N	N	Y	Y	Y	Y	Y	Y	Y	Y
Zhang et al., 2023	Y	N	Y	N	N	N	Y	Y	Y	Y	Y	Y	Y	Y
Zhang et al., 2020	Y	U	Y	N	N	U	Y	Y	Y	Y	Y	Y	Y	Y
Zhang, 2012	U	U	Y	N	U	U	Y	Y	Y	Y	Y	Y	Y	Y
Zhang et al., 2021	Y	Y	Y	Y	Y	Y	Y	Y	Y	Y	Y	Y	Y	Y
Xu, 2015	U	U	Y	N	U	U	Y	Y	Y	Y	Y	Y	Y	Y
Wu et al., 2013	Y	Y	Y	Y	Y	Y	Y	Y	Y	Y	Y	Y	Y	Y
Li & Yang, 2019	Y	N	Y	N	N	U	Y	Y	Y	Y	Y	Y	Y	Y
Lei & Zhang, 2016	Y	N	Y	N	N	U	Y	Y	Y	Y	Y	Y	Y	Y
Chen et al., 2016	U	N	Y	N	N	U	U	Y	Y	Y	Y	Y	Y	Y

Notes: Y-yes, N-no, U-unclear, NA-not applicable.

### 3.3 Descriptions of studies

#### 3.3.1 Study characteristics

All included studies were RCTs published between 2013–2024. All included RCTs were conducted in China and all but one were written in Chinese. Fifty percent of the studies were published in the last seven years (between 2018–2024), with the remaining 50% being published in the four years prior (2013–2016). Sample sizes ranged from 46 to 289 with a total participant pool of 1 124, of which 449 participants received a control intervention and 675 received an investigational medicinal product (IMP). CHM used for treatment of dizziness differed between studies, as did control methods, with some studies using a placebo and others using experimental or positive controls. Dizziness inclusion criteria varied across the studies (Table S1) as did duration of treatment which ranged from 10 d to 16 w. Due to the wide range of differences in comparators, interventions, outcomes, and study designs, statistical testing such as *meta*-analysis and heterogeneity testing was inappropriate, and these tests were not conducted. Baseline comparability of participants was recorded and accounted for in all included studies. Detailed characteristics of the included studies can be seen in Table 2. A comparison of all the herbs in each formulation can be seen in Table S3.

**Table 2:** Characteristics of included studies.

Included studies	Population	Intervention	Sample size	Age	Gender (*n*)(M/F)	Safety and tolerability	Outcome measures
Zhang et al., 2023	Posterior circulation ischemia (PCI) vertigo	Shenxiang Suhe *vs* aspirin tablets 0.7 Grams once (LD) or twice (HD) daily *vs* 100 mg/d	*n* = 120 Low-dose (LD) *n* = 40; High-dose (HD) *n* = 40; Control (Con) *n* = 40	40−85	LD: 15/40 HD: 22/40 Control: 16/40	No significant difference between groups in the blood tertiary system, liver function, and renal function.	1. Efficacy rates (%): LD: Tr: 2 w = 50%; 4 w = 60%; HD: Tr: 2 w = 63%; 4 w = 63%; Con: 2 w = 30%; 4 w = 43%. 2. Basal arterial flow: Con: BT = 39.6 ± 17.76; 2 w = 43.40 ± 8.51; 4 w = 48.40 ± 6.91. LD: BT = 39.02 ± 24.46; 2 w = 48.30 ± 12.10; 4 w = 53.88 ± 10.32. HD: BT = 40.12 ± 22.68; 2 w = 52.45 ± 12.54; 4 w = 58.30 ± 12.70. *P* ≤ 0.001 between groups. 3. Hemorheology changes for blood viscosity, fibrinogen, TC, TG, LDL, CRP, Hct.
Hao, Wang, Xie, Wang, & Zhang, 2024	PCI vertigo	Qishen Zhixuan Decoction *vs* Ginkgo only. The decoction was prepared with 400 mL water daily. Both groups received 25 mL of Ginkgo biloba leaf extract injection intravenously for two weeks.	*n* = 97 IMP: 49 Con: 48	41−80	IMP: 27/22 Con: 25/23	No abnormal liver and kidney function occurred in either group before or after treatment.	1. Efficacy rates (%): IMP: 2 w = 96% Con: 2 w = 78% 2. Dizziness scores: IMP: BT = 4.81 ± 1.08; 2 w = 1.94 ± 0.89; Con: BT = 4.83 ± 1.00; 2 w = 2.87 ± 1.31; *P* ≤ 0.001 between groups. 3. DHI: IMP: BT = 78.72 ± 9.69; 2 w = 28.68 ± 8.60; Con: BT = 79.39 ± 7.04; 2 w = 43.48 ± 13.26; *P* ≤ 0.05 between groups. 4. Fatigue: IMP: BT = 2.67 ± 0.52; 2 w = 0.72 ± 0.65; Con: BT = 2.61 ± 0.54; 2 w = 1.09 ± 0.81; *P* ≤ 0.05 between groups.
Zhang et al., 2020	Dizziness	Hu Zhang Ye *vs* manual repositioning treatment only disappears. Two cycles (four weeks).	*n* = 70 IMP: 35 Con: 35	18−75	IMP: 15/20 Con: 23/12	None reported.	1. DHI mean change: IMP: BT = 82.63 ± 5.06; 2 w = 43.43 ± 3.48 (*P* ≤ 0.01). 4 w = 35.20 ± 4.50 (*P* ≤ 0.01 *vs* BT; *P* ≤ 0.01) Con: BT = 83.40 ± 3.24; 2 w = 52.34 ± 4.61 (*P* ≤ 0.01 *vs* BT). 4 w = 40.91 ± 4.48 (*P* ≤ 0.01 *vs* BT).
Zhang et al., 2012	Dizziness	Siwu Decoction *vs* flunarizine hydrochloride only. Dose 5−10 mg before bedtime for four weeks.	*n* = 46 IMP: 26 Con: 20	26−64	Not stated.	No adverse reactions.	1. Mean total number vertigo episodes: IMP: BT = 6.14 ± 3.12; 4 w = 1.15 ± 1.36 (*P* ≤ 0.05) Con: BT= 6.37 ± 3.23; 4 w = 2.68 ± 3.22 (*P* ≤ 0.05) 2. Mean total number vertigo days: IMP: BT = 10.34 ± 8.12; 4 w = 0.97 ± 2.14 (*P* ≤ 0.05) Con: BT=10.5 ± 8.54; 4 w = 2.75 ± 3.66 (*P* ≤ 0.05). 3. Mean severity of vertigo: IMP: BT = 2.85±0.67; 4 w = 0.71±0.27 (*P* ≤ 0.05) Con: BT = 2.78±0.46; 4w = 1.34±1.46 (*P* ≤ 0.05) 4. % recurrence rate: IMP = 0%; Con = 25%.
Zhang et al, 2021	Dizziness	Ejiao *vs* placebo. 6 g/d for 56 days.	*n* = 181 IMP: 119 Placebo: 62	18−60	Not stated.	A total of 11 AEs occurred during Ejiao treatment: Anabrosis (*n* = 5); facial acne (*n* = 3). No moderate or severe AEs were reported.	1. Mean symptoms of dizziness: IMP: 0 d = Mild 53.7%; Mod 41.7%; Severe 4.6%; *P* = 0.647; 28 d = Mild 84.3%; Mod 15.7%; Severe 0%; *P* = 0.918; 56 d = Mild 93.5%; Mod 6.5%; Severe 0%; *P* = 0.043. Placebo: 0 d = Mild 56.4%; Mod 41.8%; Severe 1.8% 28 d = Mild 83.6%; Mod 16.4%; Severe 0%; 56 d = Mild 83.6%; Mod 14.5%; Severe 1.8%. 2. Secondary outcome (SF-36) Physical treatment: 28d (*P* < 0.05); Vitality treatment: 28 d (*P* < 0.01); 56 d (*P* < 0.05); Social treatment: 28 d (*P* < 0.01); Emotional: 28 d (*P* < 0.05); Health treatment: 28 d (*P* < 0.01). 3. Secondary outcome measure Haematocrit treatment: IMP: Mean change = 0.57±0.21 (*P* = 0.036); Placebo: Mean change= −1.35 ± 0.27. RBC: IMP: Mean change = −0.043 ± 0.02 (*P* = 0.048); Placebo: Mean change = −0.13 ± 0.029.
Xu, 2015	Dizziness	Pinggan Dingxuan Tang *vs* Gastrodin and Danshen Chuan Xiongqin. Injection 10 mL (BID), mixed glucose or 500 mL saline. Once a day, 10 d.	*n* = 130 IMP: 68 Con: 62	21−70	Not stated.	None reported.	1. Mean change in diagnostic and therapeutic efficacy criteria of TCM diseases (Table S2). IMP: Cure = 45.58%; Marked effect = 47.05%; Effective = 7.36%; Ineffective = 0%; Cure rate = 45.58%; Total effective rate = 92.64%. Con: Cure = 29.03%; Marked effect = 32.25%; Effective = 29.03%; Ineffective = 9.67%; Cure rate = 29.03%; Total effective rate = 61.29%.
Wu et al., 2013	CCCI	YXQNG *vs* nimodipine with placebo. 10 mg TID for eight weeks.	*n* = 289 IMP: 140 Con: 133	> 30	IMP: 52/88 Con: 58/75	AEs; 8.57% (12/140) forYXQNG group and 6.02% (8/133) for nimodipine group (*P* = 0.245). Safety and tolerability were similar between the two groups. AEs were mild to moderate in intensity and resolved without discontinuation of IMP.	1. Mean change VAS scores Headache stage rating (*P =* 0.052); Dizziness rating: BT: *P* = 0.086 8 w: *P* = 0.166 2. Heavy-headed feeling (*P =* 0.823); 3. Sleep disorder: BT: *P=*0.127; 8 w: *P* = 0.528.
Li & Yang, 2019	Vertigo	Huatan Huoxue Tang *vs* flunarizine hydrochloride 10 mg nocte and 12 mg tablets TID for 16 weeks.	*n* = 98 IMP: 49 Con: 49		IMP: 17/32 Con: 14/35	None reported.	1. DHI: 0 w: *P* = 0.668; 1 w: *P* = 0.743; 2 w: *P* = 0.260; 3 w: *P* = 0.015; 4 w: *P* = 0.314; 8 w: *P* = 0.225; 12 w: *P* = 0.200; 16 w: *P* = 0.203. 2. TCMSS: 0 w: *P* = 0.904; 1 w: *P* = 0.989; 2 w: *P* = 0.206; 3 w: *P* ≤ 0.001; 4 w: *P* = 0.016; 8 w: *P*= 0.001; 12 w: *P* = 0.001; 16 w: *P* = 0.001. 3. SAS: 0 w: *P* = 0.118; 1 w: *P* = 0.948; 2 w: *P* = 0.005; 3 w: *P* ≤ 0.001; 4 w: *P* = 0.008; 8 w: *P* = 0.001; 12 w: *P* = 0.001; 16 w: *P* = 0.002. 4. SDS: 0 w: *P* = 0.177; 1 w: *P* = 0.528; 2 w: *P* = 0.006; 3 w: *P* ≤ 0.001; 4 w: *P* = 0.314; 8 w*: P* = 0.004; 12 w: *P* = 0.007; 16 w: *P* = 0.014. 5. ASP: 0 w: *P* ≤ 0.974; 1 w: *P* ≤ 0.045; 2 w: *P* ≤ 0.001; 3 w: *P* ≤ 0.001; 4 w: *P* ≤ 0.001; 8 w: *P* ≤ 0.001; 12 w: *P* ≤ 0.001; 16 w: *P* ≤ 0.001.
Lei & Zhang, 2016	BPPV	Banxia Baizhu Tianma Decoction *vs* modified Epley manoeuvre. Decoction for oral administration, BID (morning and evening), 200mL each time, continuously treated for two weeks. Outcome assessments were performed at two days, two weeks and three months.	*n* = 53 IMP: 23 Con: 30	36−70	IMP: 9/14 Con: 11/19	None reported.	1. Clinical efficacy (Table S2). IMP: 2 d: Cure = 21.47%; Effective = 52.17%; Ineffective = 26.09%; Total effective rate = 73.91%; 2 w: Cure = 39.13%; Effective = 47.83%; Ineffective = 13.04%; Total effective rate = 86.96%; 3 m: Cure = 52.17%; Effective = 43.448%; Ineffective = 4.35%; Total effective rate = 95.65%. Con: 2 d: Cure = 13.33%; Effective = 46.67%; Ineffective = 40.00%; Total effective rate = 60.00%; 2 w: Cure = 33.33%; Effective = 40.00%; Ineffective = 26.67%; Total effective rate = 73.33%; 3 m: Cure = 36.67%; Effective = 46.66%; Ineffective = 16.67%; Total effective rate = 83.33%. 2. Comparison of blood lipid levels at three months. TG: *P* = 0.004; TC: *P* = 0.000; LDL: *P* = 0.010; HDL: *P* = 0.521; APO-A1: *P* = 0.002; APO-B: *P* = 0.043.
Chen et al., 2016	Dizziness	Jichuan Decoction plus betahistine hydrochloride *vs* betahistine hydrochloride alone Decoction (1 bag) is taken 30 min before meals and betahistine hydrochloride (1 tablet) after meals in the morning and evening. For the control group, one tablet was given TID after meals. The treatment duration was four weeks.	*n* = 40 IMP: 20 Con: 20	30−58	IMP: 12/8 Con: 10/10	Adverse reactions in both groups of patients were observed. Number and severity of AEs not reported.	1. Percentage change in Clinical Diagnosis and Therapeutic Effect Criteria (see supplemental material). IMP: 4 w: Significant effectiveness 40%; effective 50%; ineffective 10%; total effective rate 90.0% (*P* ≤ 0.05). Con: 4 w: Significant effectiveness 10%; effective 40%; ineffective 50%; total effective rate 50.0%.

Notes: IMP-treatment; Con-control; IMP-investigational medicinal product; M-male; F-female; *n*-number; BID-twice a day; TID-three times a day; TCM-traditional Chinese medicine; BT-before treatment; G-grade; d-days; w-weeks; m-months; SAS-self-rating anxiety scale; SDS-self-rating depression scale; ASP-autonomic symptom profile; TG-total serum triglycerides; TC-total cholesterol; LDL-low-density lipoprotein; HDL-high-density lipoprotein, APO-A1-apolipoprotein A1; APO-B-apo-lipoprotein B; AE-adverse event. ABF-arterial blood flow; VAS-visual analogue scale.

#### 3.3.2 CHM treatments

Eight different combinations of CHM and two single CHM were used throughout ten RCTs. The combinations included: Siwu Tang (Four ingredients Decoction), Pinggan Dingxuan Tang (decoction for calming the liver and stopping vertigo), Yangxue Qingnao Granules (granules for nourishing blood and refreshing the brain), Huatan Huoxue Tang (decoction for resolving phlegm and activating blood), Banxia Baizhu Tianma Tang (*Pinelliae Rhizoma*, *Atractylodis Macrocephalae Rhizoma* and *Gastrodiae Rhizoma* Decoction), Shenxiang Suhe Pill (Styrax Pill), Qishen Zhixuan Decoction and Jichuan Jian (decoction for replenishing fluid). The two single CHM used were *Asini Corii Colla* (Ejiao in Chinese) and *Reynoutriae Japonicae Folium* (tiger cane leaf, Huzhangye in Chinese). More detail on CHM combinations, their ingredients and mechanism of action can be found in Table S2–S4. A comparison of the actions ascribed to each herbal medicine from a WHM and a CHM perspective is provided in Table S4.

#### 3.3.3 Effects of interventions and main CHM action

All RCTs included in this review individually showed CHM to be an effective intervention in the treatment of dizziness, however due to the high heterogeneity among studies, overall effectiveness cannot be determined (Table 2). Efficacy criteria is in Table S2.

TCM philosophy adheres to an individualised medical approach, as such, all studies in this review used TCM diagnostic criteria and approached the treatment of dizziness from a different TCM aetiology. Therefore, the herbs used, and mechanisms of action were different across and within studies as shown in Table 3. Some key details such as processing methods, dosage units, and the basis for prescription modification were not consistently reported across the original studies. To address these gaps, future use of the Consolidated Standards of Reporting Trials-Chinese Herbal Medicine (CONSORT)-CHM 2017 checklist should be adopted during design and reporting of RCTs involving TCM (Cheng et al., 2017). Its use would help ensure that essential methodological criteria are met, thereby improving quality of future research.

**Table 3:** CHM actions.

Combinations of CHM/single CHM	Pathogenesis/TCM theory	TCM actions	Dosage and preparation method	Reference(s)
SXSHP	Deficiency of *qi* and blood circulation. In the theories of TCM, vertigo is related to phlegm retention and *fire* in the body drives phlegm to move. It is also believed that dizziness happens when blood stasis stops accumulating and the blood rushes up.	SXSHP is a Chinese patent medicine widely used to treat coronary heart disease and angina pectoris. It has the effect of promoting *qi*, warming, clearing away turbidity, and activating blood circulation.	0.7 g of SXSHP (Hangzhou Hu Qingyutang, National Drug Approval Z33020141) was taken orally once daily for low-dose group and twice daily for high-dose group.	Zhang et al., 2023
Qishen Zhixuan Formula	TCM believes that this disease is related to the liver, spleen and kidney, and the pathogenesis is mixed with deficiency and excess. It is mostly treated from the perspective of “*wind*, *fire*, phlegm, deficiency and stasis”.	Strengthening the spleen and replenishing *qi*, nourishing blood and symptoms: Fatigue, palpitations, spontaneous sweating, insomnia and frequent dreams, poor appetite, activating blood circulation.	Herbs were decocted in water and 400 mL of juice was taken warm in the morning and evening.	Hao, Wang, Xie, Wang, & Zhang, 2024
Huzhangye Capsule	Liver *yang* hyperactivity.	Dispelling pathogenic *wind* and eliminating phlegm, and suppressing hyperactive liver and subsiding *yang.*	Two capsules, TID after meals with 1-hour interval for 4 w.	Zhang et al.,2020
Siwu Tang	Deficiency of *qi* and blood and insufficiency of kidney essence.	*Chuanxiong Rhizoma* in Siwu Tang promotes blood circulation and *qi* movement, dispels *wind*, and relieves pain; *Angelicae Dahuricae Radix* and *Angelicae Sinensis Radix* dispel *wind*, release exterior, relieve pain, and reduce inflammation; *Pheretima* clears heat, calms the liver, and promotes meridian circulation.	Taking Siwu Tang once daily or in two divided doses for 4 w.	Zhang, 2012
Ejiao	Blood deficiency.	Tonifying the blood. Nourishing the blood and stoping bleeding. Nourishing and moistening *yin*. Moistening the lungs and large intestine.	Ejiao in a capsule taken once daily for 8 w.	Zhang et al., 2021
Pinggan Dingxuan Tang	Liver *yang* syndrome type of vertigo.	Liver calming.	Soaking the herbs in 600 mL of water for half an hour, boiling in water for 30 min, taking 300 mL of juice, boiling again with 500 mL of water, taking 200 mL of juice, mixing the two juices, and taking twice a day. Ten days make one course of treatment.	Xu, 2015
YXQNG	Blood deficiency and excessive liver *yang*. CCCI diagnosis.	Tonifying blood and clearing liver heat.	One 4 g package was given three times a day for 8 w.	Wu et al., 2013
Banxia Baizhu Tianma Tang	Treatment of BPPV, through treating for *wind*-phlegm dizziness.	Drying dampness and transforming phlegm, calming the liver and extinguishing *wind*.	Decoction for oral administration, twice a day (morning and evening), 200 mL each time for 2 w.	Lei & Zhang, 2016
Huatan Huoxue Prescription	Stagnation of turbid phlegm in middle energizer and obstruction of the orifices by blood stasis.	Classic prescription of phlegm.	All ingredients were soaked in water for 1 h, then decocted with 200 mL of water twice in the morning and evening separately and were administered warm for 16 w.	Li & Yang, 2019
Jichuan Decoction	Excessive *yang fire*, which can create phlegm, assist *yang* in transforming into *wind*, and disturb the clarity of the mind, burning of body fluids, leading to dryness and obstruction of the gastrointestinal tract.	Promoting bowel movement and moistening the intestines.	The treatment group is given one bag of decoction before meals and one tablet of Betahistine after meals for 4 w.	Chen et al., 2016

One RCT found tiger cane leaf lowered blood pressure and improved symptoms such as dizziness and headaches in hypertensive patients with liver *yang* hyperactivity. TCM theory suggests this effect was due to dispelling pathogenic *wind* and eliminating phlegm, and suppressing a hyperactive liver and subsiding *yang* (Zhang et al., 2020). A possible physiological mechanism that may relate to the effect observed is the improvement of circulation in the inner ear vestibule, which has become dysregulated as a result of high blood pressure perceived to be due to liver *yang* hyperactivity (Zhang et al., 2020).

The Siwu Tang combined with flunarizine was found to have a good therapeutic effect on migraine vertigo by addressing deficiency of *qi* and blood and insufficiency of kidney essence, while the Ejiao in isolation, significantly improved dizziness symptoms compared to placebo by tonifying the blood, stopping bleeding, nourishing and moistening *yin* and moistening the lungs and large intestine (Zhang et al., 2021, Zhang, 2012). The Pinggan Dingxuan Tang combined with acupuncture found a statistically significant difference in improving liver *yang* hyperactivity type dizziness, while the Yangxue Qingnao Granules were found to be useful in the long-term management of dizziness as a result of CCCI through tonifying the blood and clearing liver heat (Wu et al., 2013, Xu, 2015), Lei found that the Banxia Baizhu Tianma Tang, combined with manipulation therapy had significant clinical efficacy in treating dizziness caused by BPPV, and improved the blood lipid levels of participants through drying dampness and transforming phlegm, calming the liver and extinguishing *wind* (Lei & Zhang, 2016). The Huatan Huoxue prescription, a traditional combination of CHM for phlegm obstruction, was found to be effective in treating stagnation of turbid phlegm in middle energizer and obstruction of the orifices by blood stasis, resulting in significant improvement of vertigo symptoms, as well as improving overall QoL, anxiety, depression, and autonomic stimulation symptoms, while Chen found Jichuan Decoction promotes bowel movement and moistens the intestines, leading to improvement in dizziness symptoms and reducing the frequency of dizziness attacks (Chen, Guo, & Xu, 2016; Li & Yang, 2010). Zhang found that Shenxiang Suhe Pill (SXSHP) increased *fire* within the body, thereby helping to drive phlegm and stop blood stasis (Zhang et al., 2023). Hao found Qishen Zhixuan Formula hada strengthening effect on the spleen which replenishes *qi* and nourishes the blood assisting to reduce dizziness (Hao, Wang, Xie, Wang, & Zhang, 2024).

#### 3.3.4 Reduction of dizziness

Acupuncture administered in conjunction with Pinggan Dingxuan Tang resulted in a cure rate of 45.58% and a total effective rate of 92.64% according to the Diagnostic and Therapeutic Efficacy Criteria of Traditional Chinese Medicine Diseases, while the control treatment of Gastrodin injection combined with Danshen Chuanxiongqin injection resulted in a cure rate of 29.03% and total effective rate of 61.29% according to the same criteria (Xu, 2015). Alternatively, Siwu Tang combined with flunarizine resulted in a 0 recurrence of dizziness compared to flunarizine alone which showed a recurrence rate of 25% (Zhang et al., 2012).

#### 3.3.5 Dizziness free days

The Siwu Decoction combined with flunarizine hydrochloride showed a statistically significant (*P* ≤ 0.05) overall reduction in total number of vertigo episodes and a statistically significant (*P* < 0.05) increase in dizziness free days when compared to flunarizine hydrochloride alone (Zhang et al., 2012).

#### 3.3.6 Responder rate

The majority of studies used for this literature review found over 90% of participants responded to CHM with the total effective rate of the treatment group using Jichuan Decoction combined with betahistine being 90% (Chen, Guo, & Xu, 2016). In the Lei & Zhang paper, a total effective rate of 91% was found utilising two days of modified Epley repositioning procedure combined with modified Banxia Baizhu Tianma Decoction (Lei & Zhang, 2016). Again, the effective rate of the TCM group utilising self-made Huatan Huoxue prescription was 96% (Li & Yang, 2019). Xu found 91% of participants responded to CHM combined with acupuncture, while in the Zhang paper, 100% of participants responded to Siwu decoction (Zhang, 2012, Xu, 2015). The TCM combination SXSHP (0.7 g once or twice daily) showed a 30% or more improvement in their dizziness symptoms in 60% of participants at 28 d when taken once daily and 63% of participants when taken twice daily (Zhang et al. 2023), demonstrating the lowest response rate, and Qishen Zhixuan Decoction in one study showed that 96% of participants responded (Hao, Wang, Xie, Wang, & Zhang, 2024). The responder rate for Wu (Wu et al., 2013), Zhang 2021 (Zhang et al., 2021) and Zhang 2020 (Zhang et al., 2020) are unknown.

#### 3.3.7 Dizziness severity

The Siwu Decoction combined with flunarizine hydrochloride showed a statistically significant (*P* ≤ 0.05) reduction in mean severity of vertigo when compared to flunarizine hydrochloride alone (Zhang et al., 2012). Ejiao at 6 g per day was shown to reduce dizziness severity compared to placebo with 93.5% of participants rating their symptoms as mild, 6.5% moderate and 0% severe at 56 d compared to placebo in which participants rated their symptoms as mild 83.6%, moderate 14.5% and severe 1.8% at 56 d (*P* = 0.043) (Zhang et al., 2021).

The use of YXQNG at 4 g four times daily resulted in a statistically significant reduction in headache rating compared to nimodipine 10 mg (*P* = 0.052), however insignificant changes were seen in 8-week timepoint VAS scores of dizziness rating (*P* = 0.166); heavy-headed feeling (*P* = 0.823) and sleep disorder (*P* = 0.528) (Wu et al., 2013).

Huatan Huoxue prescription showed statistically significant improvements in mean TCM syndrome score scale at 3 w (*P* ≤ 0.001); 4 w (*P* = 0.016); 8 w (*P* = 0.001); 12 w (*P* = 0.001) and 16 w (*P* = 0.001) when compared to flunarizine hydrochloride capsules and betahistine mesilate tablets (Li & Yang, 2019).

Lei found Banxia Baizhu Tianma Formula to be more effective in treating dizziness than modified Epley manoeuvre with CHM having a total effective rate of 73.91% at 2 d; 86.96% at 2 w and 95.65% at 3 m according to the clinical efficacy standards, while the modified Eply manoeuvre showed a total effective rate of 60%, 73.33% and 83.33% across the same timepoints (Lei & Zhang, 2016).

Chen used the percentage change in Clinical Diagnosis and Therapeutic Effect Criteria to assess effectiveness of Jichuan Decoction combined with tablets containing betahistine hydrochloride in the treatment of dizziness compared to betahistine hydrochloride alone (Chen, Guo, & Xu, 2016). Jichuan Decoction combination treatment showed a statistically significant (90% total effective rate) improvement in dizziness compared to control (50% total effective rate; *P* ≤ 0.05) (Chen, Guo, & Xu, 2016).

No outcome of change in VAS score for dizziness or vertigo at 2 h was recorded in any RCT treatment group included in this review. No outcome on changes in nausea were recorded across any RCT included in this review.

#### 3.3.8 QoL

The DHI was used to assess reduction in dizziness and improvement in QoL in two of the studies included in this review (Li and Yang, 2019, Zhang et al., 2020). Zhang found Huzhangye Capsules combined with manual repositioning treatment resulted in a statistically significant (*P* ≤ 0.01) reduction in DHI scores at 2 and 4 w of treatment compared to manual repositioning only (Zhang et al., 2020). Li found consistently lower mean DHI scores across multiple timepoints during a 16-week treatment period utilising Huatan Huoxue prescription compared to flunarizine hydrochloride capsules and betahistine mesilate tablets (Li & Yang, 2019). DHI was also assessed by Hao demonstrating a statistically significant between group difference in favour of the active treatment at 2 w, *P* ≤ 0.05 (Hao, Wang, Xie, Wang, & Zhang, 2024).

Ejiao prescribed at 6 g/d was shown to improve QoL compared to placebo (Zhang et al., 2021). Statistically significant (*P* < 0.05) changes were seen in outcome measures on the SF-36 across all aspects at 28 d of treatment and statistically significant (*P* ≤ 0.05) changes in vitality were seen at day 56 when compared to placebo (Zhang et al., 2021).

Statistically significant (*P* < 0.05) reduction in autonomic symptoms was seen across all timepoints in the 16-week study comparing Huatan Huoxue prescription to flunarizine hydrochloride capsules combined with betahistine mesilate tablets in the treatment of vertigo (Li & Yang, 2019).

The self-assessed anxiety scale (SAS) and depression scale (SDS) were used to assess mental health and QoL in a trial comparing Huatan Huoxue prescription to flunarizine hydrochloride capsules and betahistine mesilate tablets in the treatment of vertigo (Li & Yang, 2019). Statistically significant reduction in SAS scores were seen in the treatment group at 2, 3, 4, 8, 12 and 16 w (*P* ≤ 0.05), while statistically significant reduction in SDS scores were seen in the treatment group at 2, 3, 8, 12 and 16 w (*P* ≤ 0.05) (Li & Yang, 2019).

#### 3.3.9 Haematological changes

There are additional results from Zhang on blood flow velocity, viscosity, and other haematological parameters (Zhang et al., 2023). Zhang found that the average blood flow velocity of the vertebral artery and basilar artery in all the three groups showed an upward trend from baseline (*P* ≤ 0.05) and the blood viscosity and levels of fibrinogen, haematocrit, and CRP showed a downward trend (*P* ≤ 0.05) (Zhang et al., 2023).

#### 3.3.10 Rescue medication used

None of the RCTs used for this review stated use of rescue medication at any time point.

### 3.4 Safety and tolerability

Overall safety and tolerability of CHM in the treatment of dizziness and vertigo were high. There were no statistically significant differences in adverse events (AEs) between treatment and control groups across all studies. AEs that did occur were mild to moderate in nature and involved the digestive system, nervous system or integumentary system. Some AE data was absent in the RCTs including incidence rates, AE types and handling methods of AEs, meaning comprehensive safety analysis is not possible. Future use of CONSORT-CHM 2017 checklist when designing and reporting future RCTs involving CHM would ensure accurate AE reporting and safety analysis is possible.

### 3.5 Comparison of CHM to WHM

To ascertain the differences and similarities of the CHM diagnostics of the herbal medicines from these studies compared to WHM, each herb has been compared in Table S4.

## 4 Discussion

This literature review identified RCTs that were all conducted in China, and many were conducted within the hospital setting utilising integrated Chinese and Western medicine, with each study assessing aetiology of dizziness from a Chinese medicine perspective. TCM broadly classifies the pathogenesis of vertigo as being caused by an imbalance or a stagnation of one or more of pathological products such as *wind*, *fire*, phlegm, and deficiency of *qi* and blood (Zhang et al., 2012). According to Chen, dizziness can be classified in TCM as deficient or replete, referring to deficiency of *qi*, blood, *yin*, and *yang*, and replete referring to diseases caused by a combination of *wind*, *fire*, phlegm, and stasis (Chen, Guo, & Xu, 2016).

As an example of the contrast between TCM diagnostic criteria and Western diagnostic practices, this study wants to highlight the Li & Yang paper, where participants were diagnosed according to TCM diagnostic criteria (Xia et al., 2013, Li and Yang, 2019). The Li & Yang paper investigated dizziness as a result of turbid phlegm in middle energizer and obstruction of the orifices by blood stasis, which TCM syndrome differentiation criteria identifies as follows:

(1) Stagnation of turbid phlegm in middle energizer. The main symptoms are heaviness of the head and a visual sense of spinning of objects. Accompanying symptoms could include chest tightness, nausea, vomiting, sputum, abdominal and epigastric fullness, loss of appetite, and listlessness. TCM physical examination reveals a plump and swollen tongue, with dental impressions on the edges, and white greasy coating, with a thready and slippery pulse.

(2) Obstruction of the orifices by blood stasis. The main symptoms are frequent vertigo and intense headache, such as stabbing pain. The accompanied symptoms could include dark face, purple dark tongue and lips, scaly skin, amnesia, palpitation, insomnia, tinnitus, and deafness. The tongue can look purplish and dark, with petechiae and ecchymosis. The pulse can be wiry and choppy, or small and choppy.

In contrast, Western medicine has a range of physiological diagnoses tied to different forms of dizziness that all have a clear physiological or pathological basis (Tarnutzer et al., 2025). If there is no clear reason for the dizziness, then it is often simply stated as dizziness of “unknown origin”. The reason that it is important to differentiate between the TCM diagnosis, and the Western perspective is because the treatment is varied according to the diagnosis. From the studies we identified, a variety of mechanisms were used to help the dizziness. In Chen paper, dizziness caused by excessive *yang fire*, can create phlegm, transform *yang* into *wind*, disturbing clarity of mind and creating dizziness, irritability, and anger. It can also lead to dryness and obstruction of the gastrointestinal tract. The biological basis of this being decreased secretory function of the intestinal mucosal glands and increased absorption of consumed fluid leading to constipation. Therefore, by promoting bowel movement and moistening the intestines, Chen found dizziness was effectively reduced (Chen et al., 2016). In contrast, Hesketh, et al. didn’t use a Chinese mechanism but looked at both Western and Eastern diagnosis criteria (Hesketh & Zhu, 1997). The Chinese criteria included white and greasy tongue coating, stringy and smooth pulse, diagnosed as phlegm-damp obstruction. The other studies again looked at different mechanisms, but the overall treatment as stated above was about moving the stagnation of phlegm and obstruction of orifices.

The reason moving the stagnation of phlegm and the obstruction of orifices can help dizziness via the Chinese philosophy that if energy is not flowing smoothly, like stagnant water in a pond, it can lead to conditions like dizziness. Internal phlegm stagnation arises when damp is not cleared properly and disrupts the body’s fluid metabolism allowing it to stagnate for a period of time, which in turn, can affect spleen *qi*. This stagnation can cause fogginess, cloudiness or feeling of ‘cotton wool’ in the head as well as cognitive impairment which can be linked to dizziness (Greenwood-Van Meerveld, Johnson, & Grundy, 2017). By clearing the stagnation, the fluid in the body moves smoothly, therefore relieving the pressure and stagnation in the head and reducing the dizziness.

Other TCM aetiologies of dizziness included in this review investigated relieving dizziness from the perspective of BDS, which, biologically is associated with reduced haemopoietic function, reduced white blood cell, red blood cell and platelet count potentially caused by myelosuppression, and hypo visceral dysfunction (Zhang et al., 2021). Zhang found effective treatment of BDS associated dizziness using Ejiao, known to tonify blood and nourish *yin* through pharmacological mechanisms such as increasing haematopoiesis. This effect may bedue to thecomposition of Ejiao, which includes collagen, peptides and polysaccharides (Zhang et al., 2021), and the actions of Ejiao, such as reducing liver-*yang* hyperactivity (Xu, 2015); therefore preventing liver-*yang* hyperactivity from turning to pathogenic *wind* and phlegm, as discussed in more detail above (Zhang et al., 2020).

Regarding Western medicine examinations used in this study, all relevant objective physical examinations and laboratory tests of participants returned normal results, with these patients being diagnosed with vertigo of unknown origin (Li & Yang, 2019). This shows that while Western medicine has substantial efficacy in diagnosis and treatment of dizziness and vertigo of body system dysfunction, in most cases a more individualised approach, such as that of TCM pattern differentiation, is necessary to address the underlying cause of the disease, with approximately 80% of cases of vertigo classified as stemming from unknown origin from a western medicine perspective (Bösner et al., 2018).

A dizziness from a TCM perspective can have multiple aetiologies depending on the root cause, TCM treatment requires differentiating a disease pattern in order to resolve symptoms of dizziness (Ma et al., 2021). This usually requires a thorough case history, and physical examination specific to TCM such as pulse and tongue diagnosis and individualised CHM treatment depending on individual constitution and any other associated symptoms (Ma et al., 2021). In contrast Western medicine often views disease) as the same across patients and treats the condition in isolation. Diagnosis in Western medicine involves taking a case history, physical examination and laboratory testing, and treatment will often be a medication specific to the diagnosis rather than the presenting symptoms of the patient. Therefore, Western medicine approach is substantially more straightforward in relation to conducting clinical trials to assess effectiveness of treatments, as a single intervention is associated to one diagnosis. TCM theory however requires individualised and personalised medicine to be effective, as such, assessing effectiveness of many different herbal medicines in different patients for treatment of the same condition naturally becomes more difficult.

All RCTs included in this review each found CHM, in isolation or in combination with other treatments, to be effective in relieving symptoms of dizziness, however due to the vast differences across included RCTs in CHM used, differing controls and varied measures of effectiveness, overall effectiveness of CHM in the treatment of dizziness cannot be concluded. Different processing methods of CHM are thought to have different therapeutic effects according to TCM theory, while WHM theory applies actions to botanical names of herbs, regardless of herbal processing method. This can be seen clearly when comparing CHM actions to WHM actions ascribed to the herbs used across the included studies (Table S5).

CHM offers a unique treatment option for dizziness that looks at the condition differently to standard Western diagnostics. These studies show that CHM could play a pivotal role in helping people with dizziness, however, further studies are required to confirm these results. Combining both standard Western diagnostics with Chinese diagnostics and assessing the outcomes with Western treatment options and CHM options in the same individual will give further insight into how CHM maybe used to assist dizziness. While ensuring RCT development, conducting and reporting aligns with the CONSORT-CHM 2017 checklist will enable more reliable comparison and analysis of effectiveness.

All of the RCTs used in this literature review were translated from Chinese into English with the use of AI for data extraction, excepting one which was published in English (Zhang et al., 2023). This was considered when extracting data and conducting critical appraisal. As the translation was conducted with the assistance of AI, there could be information that was translated incorrectly. Lastly, this manuscript was primarily written by complementary medicine professionals that were not specially trained in CHM. To ensure the article was written with the understanding from CHM perspective, a first completed draft was reviewed by a CHM practitioner and academic. Due care was taken to ensure the accuracy of Pin Yin names and translations in accordance with the Chinese Medicine Board of Australia’s Guidelines. However, despite adherence to these standards, minor errors may still occur due to transliteration complexities and CHM variations. While all efforts were made to ensure a global search of RCTs involving TCM and dizziness, the RCTs that met our inclusion criteria are all conducted in China, as a result, there is a risk of regional publication bias.

A total of ten eligible studies were identified through the literature screening process. Although *meta*-analysis can be statistically feasible with as few as eight to ten studies, a quantitative synthesis was not undertaken because substantial heterogeneity existed across the included studies in terms of interventions, comparators, outcome measures, and study designs. This level of clinical and methodological heterogeneity would have rendered any pooled estimates potentially misleading and the resulting summary effect unreliable. Consequently, findings were synthesised descriptively to provide a more accurate representation of the available evidence. Future trials would benefit from greater standardisation in study design, intervention reporting, comparator selection, and outcome assessment. In particular, adoption of the CONSORT-CHM 2017 checklist in the design and reporting of randomised controlled trials would improve reporting consistency, facilitate comparisons across studies, and enhance the feasibility and reliability of future *meta*-analyses.

## 5 Conclusion

Overall, this literature review found CHM from the ten studies identified in this review to be effective in alleviating dizziness in the majority of cases, with most studies finding some evidence of effectiveness between 30% and 90% of the treated cohort. The trials all utilised individualised herbal medicine with a range of different herbs and approached the condition from the perspective of pattern differentiation. These results are insufficient to conclude any concise outcomes regarding CHM for dizziness. However, CHM has the potential to be a safe and effective long-term option in the treatment and management of patients experiencing acute and chronic presentation of dizziness, especially those of which Western medicine has diagnosed dizziness of unknown origin.

## CRediT authorship contribution statement

**Charlotte McMahon:** Data curation, Writing – review & editing. **Chris Zaslawski:** Writing – review & editing, Validation. **Vanessa Vigar:** Writing – review & editing. **Nicole Hannan:** Validation, Methodology, Formal analysis, Writing – review & editing. **Sophia Gerontakos:** Formal analysis, Writing – review & editing. **Janet Schloss:** Conceptualization, Methodology, Software, Validation, Formal analysis, Investigation, Supervision, Project administration, Funding acquisition.

## Declaration of competing interest

The authors declare that they have no known competing financial interests or personal relationships that could have appeared to influence the work reported in this paper.

## Appendix A Supplementary material

Supplementary Data 1 The supplementary material contains tables expanding on herbal medicines identified in this review.
