# Supplementary material for: Systematic review of traditional Chinese medicine in treatment of dizziness
Source: Chin Herb Med. 2026 Aug 22;18(4):926–38. doi: 10.1016/j.chmed.2026.08.008 (PMC13712348; doi:10.1016/j.chmed.2026.08.008)
Supplement: Supplementary file 1 [file mmc1.docx]

**Supplementary materials**

Table S1 Dizziness article inclusion criteria.

| Inclusion criteria | Hao, Wang, Xie, Wang, & Zhang, 2024 | Zhang et al., 2023 | Zhang et al., 2020 | Zhang, 2012 | Zhang et al., 2021 | Xu, 2015 | Wu et al., 2013 | Li & Yang, 2019 | Lei & Zhang, 2016 | Chen, Guo, & Xu, 2016 |
| --- | --- | --- | --- | --- | --- | --- | --- | --- | --- | --- |
| Ischemic vertigo symptoms of posterior circulation |  | X |  |  |  |  |  |  |  |  |
| Patients with transcranial duplex (TCD) sonography indicating abnormal hemodynamics of the vertebrobasilar artery system |  | X |  |  |  |  |  |  |  |  |
| Patients with presence of atherosclerotic plaques, lumen stenosis, and occlusion in the vertebrobasilar artery system confirmed by color Doppler ultrasound |  | X |  |  |  |  |  |  |  |  |
| Patients with the presence or absence of brainstem, cerebellum, and occipital lobe infarction diagnosed using a head computed tomography (CT) |  | X |  |  |  |  |  |  |  |  |
| Patients were diagnosed with posterior circulation ischemic central vertigo in accordance with the diagnostic criteria (Chinese Medical Education Association Vertigo Professional Committee, 2020) | X |  |  |  |  |  |  |  |  |  |
| Traditional Chinese medicine (TCM) diagnosis and syndrome differentiation criteria refer to the diagnostic criteria for vertigo and the syndrome differentiation criteria for *qi* and blood deficiency in the “22 specialties and 95 diseases diagnosis and treatment programs of traditional Chinese medicine” | X |  |  |  |  |  |  |  |  |  |
| Meet both Chinese and Western diagnostic criteria and TCM syndrome differentiation criteria | X |  |  |  |  |  |  |  |  |  |
| Medical history supporting the diagnosis of benign paroxysmal positional vertigo (BPPV) |  |  | X |  |  |  |  |  |  |  |
| Diagnosis of BPPV was based on the diagnostic criteria (Bhattacharyya et al., 2008) |  |  | X |  |  |  |  |  |  |  |
| Neuhauser diagnostic criteria |  |  |  | X |  |  |  |  |  |  |
| Vertigo attacks at least twice a month on average in the last three months or had more than four days of dizziness per month |  |  |  | X |  |  |  |  |  |  |
| Syndrome differentiation of TCM is blood deficiency syndrome (BDS) |  |  |  |  | X |  |  |  |  |  |
| Diagnosis of liver-*yang* hyperactivity type dizziness |  |  |  |  |  | X |  |  |  |  |
| Meeting the Diagnostic and Therapeutic Efficacy Criteria of Traditional Chinese Medicine Diseases (National Administration of Traditional Chinese Medicine, 1994) |  |  |  |  |  | X |  |  |  |  |
| Presence of one or more of these symptoms: Dizziness, especially positional dizziness, vertigo, heavy-headed feeling and/or headache |  |  |  |  |  |  | X |  |  |  |
| Presence of cerebrovascular stenosis, especially fundus oculi arteriosclerosis, revealed by transcranial doppler |  |  |  |  |  |  | X |  |  |  |
| Symptoms of vertigo | X |  |  |  |  |  |  | X |  |  |
| TCM syndrome differentiation criteria for stagnation of turbid phlegm in middle energizer and obstruction of the orifices by blood stasis |  |  |  |  |  |  |  | X |  |  |
| History of transient dizziness induced by specific head position changes, which usually occurs when the patient is sitting up or lying down |  |  |  |  |  |  |  |  | X |  |
| Nystagmus, occurring 5−15 s after position change, and the duration of dizziness and nystagmus tremor does not exceed 30 s |  |  |  |  |  |  |  |  | X |  |
| Positive for the Dix-Hallpike induced test |  |  |  |  |  |  |  |  | X |  |
| Chinese medicine diagnostic criteria |  |  |  |  |  |  |  |  | X |  |
| Diagnosis of late-stage stroke |  |  |  |  |  |  |  |  |  | X |
| Kidney *yang* deficiency, essence deficiency, clear yang not being generated, and turbid *qi* not descending syndrome in TCM |  |  |  |  |  |  |  |  |  | X |

Note: X-inclusion criteria used for correlating study.

**Table S2** Efficacy criteria used in studies.

**Diagnostic and therapeutic efficacy criteria of TCM diseases:**

The efficacy observation is classified according to the National Administration of Traditional Chinese Medicine (1994), “Diagnostic and Therapeutic Efficacy Criteria of Traditional Chinese Medicine Diseases” as follows:

Cure: dizziness and accompanying symptoms disappear, and normal work is restored;

Marked effect: dizziness symptoms basically disappear or significantly alleviate compared to before treatment, occasionally with mild dizziness symptoms, rarely accompanied by self-rotating feeling of visual objects or unsteady standing;

Effective: the degree and frequency of dizziness are reduced, but there are still slight self-rotating feelings of visual objects;

Ineffective: dizziness and other symptoms are not relieved or worsen.

**Clinical efficacy standards:**

Cure: disappearance of various positions of dizziness symptoms and positional nystagmus;

Effective: mild and transient dizziness symptoms when the head position changes, Dix-Hallpike test-induced nystagmus with shorter duration;

Ineffective: dizziness or positional nystagmus showed no significant improvement compared with before treatment, or the symptoms worsened even when the patient’s head was in different positions for a period of time.

**Clinical diagnosis and therapeutic effect criteria**:

A marked effect means that the patient’s clinical symptoms have completely disappeared after treatment, with no impact on their daily life. Effective means that the patient’s clinical symptoms have improved to varying degrees after treatment. Ineffective means that the patient’s clinical symptoms have not improved or have even worsened after treatment. The marked effect rate is calculated as the number of marked effects divided by the total number of cases, multiplied by 100%. The total effective rate is calculated as the sum of the marked effects and effective cases divided by the total number of cases, multiplied by 100%.

**Table S3** Comparison of herbs included in each of formulations.

| CHM pin yin name | Shenxiang Suhe Pills (SXSH) | Qishen Zhixuan Decoction | Huzhangye Capsules | Siwu Decoction | Ejiao | Pinggan Dingxuan Tang | Yangxue Qingnao Granules | Huatan Huoxue Prescription | Banxia Baizhu Tianma Tang | Jichuan Decoction |
| --- | --- | --- | --- | --- | --- | --- | --- | --- | --- | --- |
| Anxixiang |  | X |  |  |  |  |  |  |  |  |
| Bajitian |  |  |  |  |  |  |  |  |  | X |
| Baiguo | X |  |  |  |  |  |  |  |  |  |
| Baishao |  |  |  |  |  | X | X |  |  |  |
| Baizhi |  |  |  | X |  |  | X |  |  |  |
| Baizhu | X | X |  |  |  |  |  | X | X | X |
| Banxia |  |  |  |  |  |  |  | X | X |  |
| Bingpian |  | X |  |  |  |  |  |  |  |  |
| Chaihu | X |  |  |  |  |  |  | X |  | X |
| Chenpi | X |  |  |  |  |  |  | X | X |  |
| Cheqianzi |  |  |  |  |  | X |  |  |  |  |
| Chishao |  |  |  |  |  |  |  | X |  |  |
| Chuanniuxi |  |  |  |  |  | X |  |  |  |  |
| Chuanxiong |  |  |  | X |  |  | X | X |  |  |
| Cishi |  |  |  |  |  |  |  |  |  | X |
| Dahuang |  |  |  |  |  | X |  |  |  |  |
| Danshen | X |  |  |  |  |  |  | X |  |  |
| Danggui | X |  |  | X |  |  | X |  |  | X |
| Dangshen | X |  |  |  |  |  |  |  |  | X |
| Dazao | X |  |  |  |  |  |  |  | X |  |
| Difuzi |  | X |  |  |  |  |  |  |  |  |
| Dilong |  |  |  | X |  | X |  |  |  |  |
| Dihuang |  |  |  |  |  | X | X |  |  | X |
| Dingxiang |  | X |  |  |  |  |  |  |  |  |
| Duzhong |  |  |  |  |  |  |  |  |  | X |
| Ejiao |  |  |  |  | X |  |  |  |  |  |
| Fuling | X |  |  |  |  |  |  | X | X |  |
| Gancao | X |  |  |  |  | X |  | X | X |  |
| Gaoben |  |  |  |  |  |  |  | X |  |  |
| Gegen | X |  |  |  |  |  |  |  |  | X |
| Gouqizi |  |  |  |  |  | X |  |  |  | X |
| Gouteng |  |  |  |  |  | X |  |  |  | X |
| Heye |  |  |  |  |  |  |  |  |  | X |
| Honghua | X |  |  |  |  |  |  |  |  |  |
| Hongjingtian | X |  |  |  |  |  |  |  |  |  |
| Huzhangye |  |  | X |  |  |  |  |  |  |  |
| Huangqin | X |  |  |  |  |  |  | X |  |  |
| Huangqi | X |  |  |  |  |  |  |  | X |  |
| Jiang | X |  |  |  |  |  |  | X | X |  |
| Jili |  |  |  |  |  | X |  |  |  |  |
| Juemingzi |  |  |  |  |  | X | X |  |  |  |
| Juhua |  |  |  |  |  | X |  |  |  |  |
| Longdan |  |  |  |  |  | X |  |  |  |  |
| Luhui |  | X |  |  |  |  |  |  |  |  |
| Luobumaye |  |  |  |  |  | X |  |  |  |  |
| Mudanpi |  |  |  |  |  | X |  |  |  |  |
| Niuxi |  |  |  |  |  |  |  |  |  | X |
| Nuzhenzi |  |  |  |  |  | X |  |  |  |  |
| Renshen | X |  |  |  |  |  |  |  |  |  |
| Roucongrong |  |  |  |  |  |  |  |  |  | X |
| Ruxiang |  | X |  |  |  |  |  |  |  |  |
| Shanyao | X |  |  |  |  |  |  |  |  |  |
| Shanzhaye | X |  |  |  |  |  |  |  |  |  |
| Shexiang |  | X |  |  |  |  |  |  |  |  |
| Shengma |  |  |  |  |  |  |  |  |  | X |
| Shenqu | X |  |  |  |  |  |  |  |  |  |
| Shouwuteng |  |  |  |  |  | X |  |  |  |  |
| Shudihuang |  |  |  |  |  |  | X |  |  |  |
| Shuiniujiao |  | X |  |  |  |  |  |  |  |  |
| Suanzaoren |  |  |  |  |  | X |  |  |  |  |
| Suhexiang |  | X |  |  |  |  |  |  |  |  |
| Taoren | X |  |  |  |  |  |  |  |  |  |
| Tianma |  |  |  |  |  | X |  |  | X | X |
| Wuzhuyu |  |  |  |  |  |  |  | X |  |  |
| Xiakucao |  |  |  |  |  | X | X |  |  |  |
| Yimucao |  |  |  |  |  | X |  |  |  |  |
| Yuanzhi |  |  |  |  |  | X |  |  |  |  |
| Zexie |  |  |  |  |  | X |  | X | X | X |
| Zhenzhu |  |  |  |  |  | X | X |  |  |  |
| Zhishi |  |  |  |  |  |  |  |  |  | X |
| Zhizi |  |  |  |  |  | X |  |  |  |  |
| Zhuru | X |  |  |  |  |  |  |  |  |  |

Note: X-presence in formulation.

**Table S4** Comparison of CHM and WHM actions of herbs identified in this review.

| Chinese/pin yin name | Botanical name | Common name(s) | TCM actions | WHM actions |
| --- | --- | --- | --- | --- |
| Anxixiang | *Styrax tonkinensis* (Pierre) Craib ex Hart. | *Benzoinum*;  Benzoin | Opens the orifices, resolves phlegm and awakens the spirit invigorates the blood, promotes the movement of *qi* and alleviates pain, promotes the movement of *qi* and blood, unblocks the channels and collaterals and alleviates pain. | Not used in WHM. |
| Bajitian | *Maranda officinalis* How | *Morindae officinalis Radix*;  Morinda root | Tonifies the kidneys and strengthens *yang*. Disperses *wind*-cold-damp and strengthens sinews and bones. | Acaricidal, antidepressant, anti-fatigue, anti-inflammatory, anti-osteoporotic, fertility effects, hypoglycaemic. |
| Baiguo | *Gingko biloba* L. | *Ginkgo Semen* | Astringes the lungs, expels phlegm and stops wheezing stabilizes the lower energizer, eliminates dampness, stops discharge and astringes the urine. | Anti-inflammatory effects, anticancer effects, anticoagulant/antiplatelet effects, antidiabetic effects, antimicrobial effects, antioxidant effects, cardiovascular effects, drug metabolizing effects, lipid-lowering effects, neurologic/CNS effects, seizure threshold-lowering effects. |
| Baishao | *Paeonia lactiflora* Pall. | *Paeoniae Radix Alba*;  White peony root | Nourishes the blood and regulates menstruation. Astringes *yin* and adjusts the *ying* and *wei*. Calms liver *yang* and liver wind and alleviates pain. Softens the liver and alleviates pain (Penner, 2017). | Analgesic, anti-angiogenic, anti-cancer, antidiabetic, anti-inflammatory, anti-lipidemic, antimicrobial, antioxidant, anti-proliferative, antiviral, cognitive, DNA protective, oestrogenic, hematologic, hepatoprotective, immunologic, neuroprotective, spasmolytic, vasodilatory. |
| Baizhi | *Angelica dahurica* (Fisch ex Hoffm.) Benth. et Hook f  *A. dahurica* (Fisch ex Hoffm.) Benth et Hook f var *formosana* (Boiss) Shan et Yuan | *Angelicae Dahuricae Radix* | Expels *wind*, eliminates dampness, unblocks the nasal passages, dispels cold and alleviates pain. Reduces swelling, eliminates toxins and expels pus. Expels dampness, alleviates discharge and treats leukorrhea and diarrhea. | Anti-inflammatory, anti-tumour, anti-oxidant, analgesic, anti-viral, anti-microbial, cardio-vascular effects, nervous system effects, hepato-protective, dermatologic effects, anti-diabetic, immunomodulatory, antilipidemic (Zhao et al., 2022). |
| Baizhu | *Atractylodes macrocephala* Koidz. | *Atractylodis Macrocephalae Rhizoma* | Tonifies spleen and augments *qi*. Dries dampness and promotes water metabolism. Stabilizes the exterior and stops sweating. Calms the foetus. | Acaricidal, anti-cancer, anti-diabetic, anti-gout, anti-inflammatory, anti-platelet, anti-viral, antimicrobial, appetite stimulation, GIT effects, immunomodulator, anti-obesity. |
| Banxia | *Pinellia ternate* (Thunb.) Breit. | *Pinelliae Rhizoma* | Dried dampness and transforms phlegm. Descends rebellious *qi* and stops vomiting/harmonises the stomach. Dissipates nodules and reduces stagnation. Treats sores, skin ulcerations and carbuncles and reduces swelling. | Antimicrobial, effects on cytochrome P450 (CYP450) pathway. |
| Bingpian | *Borneolum Syntheticum* | Borneol | Aromatically opens the orifices, revives the spirit and unblocks closed disorders, clears heat, drains fire, resolves toxicity, clears the eyes and removes superficial visual obstructions clears heat, alleviates pain, dissipates nodules and alleviates itching | Not used in WHM. |
| Chaihu | *Bupleurum chinense* DC.  *B. scorzonerifolium* Willd. | *Bupleuri Radix*;  Chinese thorowax root | Resolves *shao yang* disorders and reduces fever/harmonises interior and exterior. Spreads liver *qi* and relieves stagnation. Raises *yang qi*, specifically the clear *qi* of the stomach and gall bladder. Disperses wind-heat and resolves phlegm and congestion. | Adrenal effects, antibacterial, anticoagulant and antiplatelet, antineoplastic, antioxidant, antipyretic, antispasmodic, antiviral, GIT, genitourinary, hematologic effects, hepatic effects, immunologic effects. |
| Cheqianzi | *Plantago asiatica* L.  *P. depressa* Willd. | *Plantaginis Semen*;  Plantain seed;  Blonde psyllium | Promotes urination and clears damp heat. Promotes urination to solidify the stool. Clears liver heat and clears the eyes. Clears the lungs, expels phlegm and stops coughing. | Anti-cancer, anti-diarrheal, anti-lipemic, blood urea nitrogen (BUN) effects, hypoglycaemic, irritable bowel syndrome (IBS) effects, laxative, levodopa modulator, ulcerative colitis remission, anti-obesity. |
| Chenpi | *Citrus reticulata* Blanco | *Citri Reticulatae Pericarpium*;  Dried tangerine peel | Regulates *qi*, adjusts the middle and relieves the diaphragm. Dries dampness and transforms phlegm. Helps prevent stagnation from tonic herbs. Descends *qi*. | Anti-cancer, anti-genotoxicity, anti-inflammatory, vulnerary, modulation of bone density, anti-atherosclerotic, neuropharmacological activities, antiaging, antimicrobial, antioxidant Shorbagi, Fayek, Shao, & Farag, 2022). |
| Chishao | *Paeonia lactiflora* Pall*.*  *P. veitchii* Lynch | *Paeoniae Radix Rubra*;  Red peony root | Invigorates the blood, dispels blood stasis and relieves pain. Clears heat and cools blood. Clears liver fire and relieves eye pain. Reduces swelling from sores and abscesses. | As above Bai Shao. |
| Chuanniuxi | *Cyathula officinalis* Kuan | *Cyathulae Radix*;  Medicinal cyathulae root | Wind-damp-dispelling  and blood-stasis-removing, diuretic and nourishing properties, removes blood stasis, tonifying the  liver and kidney, strengthening the bones and muscles (Huang et al., 2019). | Effects on the haematological system, effects on climacteric syndrome, effects on immune system, anti-tumour, anti-oxidant, anti-aging, effects on metabolism and growth, angio-genesis promoting (Huang et al., 2019). |
| Chuanxiong | *Ligusticum chuanxiong* Hort. | *Chuanxiong Rhizoma* | Acts on the liver, gallbladder and pericardium, invigorates blood and promotes movement of *qi* (Penner, 2017). Expels wind and alleviates pain. Predominantly used to treat headache and gynaecological diseases (Kong et al., 2024). | Anti-cerebral ischemia activity, anti-myocardial ischemia activity, vascular protective, anti-thrombotic, anti-hypertensive and vasodilator, anti-atherosclerosis, anti-spasmodic and analgesic, anti-pyretic and inflammatory, anti-cancer, anti-oxidant, anti-asthmatic, progesterone-like activity, hepato-protective, chondroprotective (Kong et al., 2024). |
| Cishi | − | *Magnetitum*;  Magnetite | Anchors liver *yang* and calms the spirit. Benefits *yin*, nourishes the kidneys, augments the liver and improves hearing and vision. Aids the kidneys in grasping *qi*. | Not commonly used in WHM. Contains many minerals and heavy metals including iron (Fe), lead (Pb), cadmium (Cd), copper (Cu), arsenic (As) and mercury (Hg). May be beneficial in treating nutrient deficiencies (Fu et al., 2011). |
| Dahuang | *Rheum palmatum L.*  *R. tanguticum* Maxim. ex Balf.  *R. officinale* Baill. | *Rhei Radix* et *Rhizoma*;  Rhubarb | Drains and purges accumulations. Drains fire, clears heat, transforms dampness and promotes urination. Drains heat from the blood. Invigorates the blood and dispels blood stasis. Clears heat and reduces fire toxicity. Clears heat and eliminates phlegm. | Anti-diarrheal, anti-inflammatory, anti-obesity, antiviral, oestrogen receptor activation, laxative, lipid-lowering, pancreatic effects, renal effects. |
| Danshen | *Salvia miltiorrhiza* Bge. | *Salviae Miltiorrhizae Radix* et *Rhizoma*;  Red sage;  Danshen root | Invigorates the blood and dispels blood stasis. Clears heat and soothes irritability. Cools the blood and reduces abscesses. Nourishes the blood and calms the spirit. | Antibacterial, anti-inflammatory, cardiovascular effects, anti-thrombotic, anti-platelet, hepatoprotective, ischemic protective, pancreatic protective effects. |
| Danggui | *Angelica sinensis* (Oliv.) Diels | *Angelicae Sinensis Radix*;  Chinese angelica;  Dong quai | Tonifies the blood and regulates the menses. Invigorates and harmonises the blood and disperses cold. Moistens the intestines and unblocks the bowels.  Reduces swelling, expels pus, generates flesh and alleviates pain. Stops coughing and treats dyspnoea. | Antiarrhythmic, anticoagulant, antihypertensive, anti-inflammatory, antineoplastic, antiplatelet, antiulcer, cardiovascular protective, dermatologic effects, genitourinary effects, hematologic effects, hepatoprotective, hormonal effects, immunologic effects, anti-spasmodic, anti-asthmatic, uterine effects. |
| Dangshen | *Codonopsis pilosula* (Franch.) Nannf.  *C. pilosula* Nannf. var. *modesta* (Nannf.) L. T. Shen  *C. tangshen* Oliv. | *Codonopsis Radix*;  Tangshen | Tonifies the middle Jiao and augments *qi*. Tonifies the lungs. Restores the constitution and assists herbs that release the exterior or drain downward when there is significant *qi* deficiency. Nourishes the blood and promotes the generation of body fluids. | Anti-cancer, anti-inflammatory, hypo-glycaemic, gastro-protective, immunomodulating effects, renal effects, adipose tissue modulation. |
| Dazao | *Ziziphus jujube* Mill. | *Jujubae Fructus*;  Chinese date | Tonifies the spleen and stomach, augments *qi*. Nourishes the blood and calms the spirit. Moderates and harmonises the harsh properties of other herbs. | Analgesic, antidiabetic, anti-inflammatory, antilipidemic, antimicrobial, antineoplastic, antioxidant, antiviral, cardiovascular effects, gastrointestinal effects, hepatoprotective, immunological activity, neuroprotective, nutritive, anti-obesity. |
| Difuzi | *Kochia scoparia* (L.) Schrad | *Kochiae Fructus*  Kochia fruit; Broom cypress; Belvedere fruit | Clears damp-heat and promotes urination expels dampness and stops itching. Clears wind-heat affecting the eyes and head. | Unknown |
| Dilong | *Pheretima aspergillum* (E. Perrier);  *P. vulgaris* Chen;  *P. guillelmi* (Michaelsen);  *P. pectinifera* Michaelsen | *Pheretima*;  Earthworm | Drains heat, settles convulsions, extinguishes wind and stops spasms. Clears heat and unblocks and promotes movement in the channels and collaterals. Clears heat and calms wheezing. Clears heats and promotes urination. Anchors liver *yang* and lowers blood pressure. Promotes healing topically. | Not used in WHM. |
| Dihuang | *Rehmannia glutinosa* Libosch. | *Rehmanniae Radix*;  Chinese foxglove root | Nourishes the blood nourishes liver and kidney *yin.* Strongly enriches *yin* and relieves wasting and thirsting disorders. Nourishes *jing* and blood and fills the marrow arrests coughing and wheezing. | Anti-allergy, anti-inflammatory, antidiabetic, antihypertensive, antitumor, drug metabolism effects, endocrine effects, gastrointestinal effects, hematological properties, immune effects, neurological effects |
| Dingxiang | *Eugenia caryophyllata* Thunb. | *Caryophylli Flos*;  Clove; Eugenia; | Warms the middle energizer, opens the stomach, relieves pain and regulates and descends *qi*. Warms the kidneys and assists yang. | Analgesic, anticancer, antimicrobial, hormonal effects, hypotensive. |
| Duzhong | *Eucommia ulmoides* Oliv. | *Eucommiae Cortex*;  Eucommia bark | Tonifies the kidneys and liver, strengthens the sinews and bones. Aids in the smooth flow of *qi* and blood. Calms the foetus. Lowers blood pressure. Tonifies kidney *yang.* | Cardio-protective, anti-hypertensive, anti-hyperlipidaemic, anti-oxidant, anti-bacterial, anti-viral, anti-inflammatory, anti-obesity, metabolic modulation, anti-tumor, neuroprotective, phyto-estrogenic, hepato-protective (He et al., 2014). |
| Ejiao | *Equus asinus* L. | Asini Corii Colla;  Donkey-hide glue;  Gelatin | Tonifies the blood. Nourishes the blood and stops bleeding. Nourishes and moistens *yin*. Moistens the lungs and large intestine. | Anti-oxidant, anti-inflammatory, bone density effects, hematopoietic effects. |
| Fuling | *Poria cocos* (Schw.) Wolf | *Poria*;  Indian bread | Promotes urination and leaches out dampness. Strengthens the spleen and harmonises the middle Jiao. Strengthens the spleen. Quiet the heart, calms the spirit, and soothes the nerves. | Anti-cancer, anti-emetic, anti-inflammatory, antioxidant, inhibit bone reabsorption, immunologic effects, nephroprotective. |
| Gancao | *Glycyrrhiza uralensis* Fisch.;  *G. inflata* Bat.;  *G. glabra* L. | *Glycyrrhizae Radix* et *Rhizoma*;  Liquorice root | Tonifies the spleen and augments *qi*. Moistens the lungs, resolves phlegm and stops coughing. Moderates and alleviates pain. Clears heats and relieves fire toxicity. Antidote for many toxic substances (internal and topical). Moderates and harmonises the harsh properties of other herbs and guides the herbs to all twelve channels. | Anti-cancer, anti-inflammatory, antimicrobial, antioxidant, antispasmodic, anti-ulcer, antiviral, cortisol effects, liver enzyme effects, hormonal effects, immunomodulatory effects, anti-hyperlipidemic. |
| Gaoben | *Ligusticum sinense* Oliv.;  *L. jeholense* Nakai et Kitag. | *Ligustici Rhizoma* et *Radix*; Chinese lovage root | Expels wind and cold. Dispels wind-dampness and alleviates pain. | Gastro-intestinal effects, anti-spasmodic, photo-toxic effects, diuretic. |
| Gegen | *Pueraria lobate* (Willd.) Ohw | *Puerariae Lobatae Radix*;  Kudzu vine root | Discharges exterior conditions and releases the muscles, especially of the neck and upper back. Relieves heat and generates fluids (relieves thirst). Vents and discharges measles. Raises spleen *yang* and stops diarrhea. Alleviates symptoms of hypertension. | Anti-inflammatory, hypo-cholesterolemic, antioxidant, antipyretic, antithrombotic, bone effects, cardio-protective, hypo-tensive, vasorelaxant, anti-arrhythmic, phytoestrogen, hypoglycaemic, neuroprotective, anti-depressant. |
| Gouqizi | *Lycium barbarum* L. | *Lycii Fructus*;  Chinese wolfberry;  Goji berry | Cools the blood and drains *yin* deficiency fire (steaming bones). Drains kidney fire, eliminates lurking heat and clears heat from the bones. Clears and drains heat in the lungs. Clears heat, cools blood and stops bleeding. | Anti-cancer, anti-diabetic, anti-fatigue, anti-microbial, anti-oxidant, anti-hypertensive, hypo-lipidemic, cardio-protective, anti-oxidant, derma-protective, fertility effects, hepato-protective, immunostimulant, ophthalmic effects. |
| Gouteng | *Uncaria rhynchophylla* (Miq.) Miq. ex Havil.;  *U. macrophylla* Wall.;  *U. hirsuta* Havil.;  *U. sinensis* (Oliv.) Havil*.*  *U. sessilifructus* Roxb. | *Uncariae Ramulus Cum Uncis*;  Gambir plant | Extinguishes wind and alleviates spasms. Drains liver heat and pacifies liver *yang*. Releases the exterior. | Antimutagenic activity, anti-oxidant, cytoprotective. (Liu, Ren, Geng, & Liu, 2026). |
| Heye | *Nelumbo nucifera* Gaertn. | *Nelumbinis Folium*;  Lotus leaf | Relieves summerheat, raises and clears spleen *yang.* Removes stagnation and stops bleeding | Anti-cancer, anti-diarrheal, anti-inflammatory, anti-obesity, antidiabetic, antimicrobial, antipyretic, antiviral, cardioprotective, dermatological effects, hepatoprotective, hormonal effects, immunomodulatory, neurological effects, sleep effects. |
| Honghua | *Carthamus tinctorius* L. | *Carthami Flos*;  Safflower; Saffron; Carthamus | Invigorates the blood, dispels blood stasis, opens the channels, unblocks menstruation and alleviates pain. | Anti-asthmatic, anti-seizure, anticancer, anticoagulant, antidepressant, antidiabetic, antioxidant, anxiolytic, cardiovascular effects, chemoprotective, erectile dysfunction protective, hypolipidemic, hepatic effects, hypotensive, immunomodulating, memory-enhancing, neuroprotective, ocular protective |
| Hongjingtian | *Rhodiola crenulate* (Hook. f. et Thoms.) H. Ohba | *Rhodiolae Crenulatae Radix* et *Rhizoma*;  All-grass of Rhodiola; Golden root; Rose-root; Aaron’s rod; Arctic root; King's crown; Lignum rhodium; Orpin rose | Fortifies the spleen and tonifies *qi.* Clears lung heat, nourishes lung *yin* and relieves cough. Invigorates the blood and transforms stasis. Promotes contraction. | Adaptogenic, anti-inflammatory, antiaging, antiarrhythmic, antibacterial, anticancer, antidepressant. antidiabetic, antihypertensive, antihypoxic, antioxidant, antiviral, anxiolytic, cardioprotective, athletic performance enhancement effects, central nervous system effects, cognitive effects, hepatoprotective, hypolipidemic, immunomodulatory, neuroprotective. |
| Huzhangye | *Polygonum cuspidatum* Sieb. et Zucc | *Polygoni Cuspidati Rhizoma* et *Radix*;  giant knotweed/Japanese knotweed | Invigorates the blood, dispels blood stasis, unblocks the channels and stops pain. Clears heat and resolves dampness. Discharges toxins and disperses swelling. Drains heat, transforms phlegm and stops cough. | Antiallergic, antibacterial, anticancer, anti-diabetic, anti-inflammatory, antilipemic, antioxidant, antiviral, cardioprotective, anti-gingivitis, phytoestrogen, immune-stimulant, laxative, neuroprotective, vulnerary, xanthine oxidase inhibition. |
| Huangqin | *Scutellaria baicalensis* Georgi | *Scutellariae Radix*;  Baical skullcap root | Clears heat and dried dampness. Drains fire and detoxifies. Cools the blood and stops bleeding. Clears heat and calms the foetus. Calms ascending liver *yang.* | Anti-allergy, anticancer, antidiabetic, anti-inflammatory, antiretroviral, uterine relaxant, hematologic effects, immunomodulation, lipid modulation effects, neuroprotective effects, BPH inhibition, sedative. |
| Huangqi | *Astragalus membranaceus* (Fisch.) Bge. var. mongholicus (Bge.) Hsiao;  *A. membranaceus* (Fisch.) Bge. | *Astragali Radix*;  Astragalus root;  Milk-vetch root | Tonifies *qi* and blood, strengthens the spleen and raises the *yang* *qi* of the spleen and stomach. Tonifies *wei qi*, stabilizes the exterior and tonifies the lungs (aids the circulation of moisture downward from the face). Promotes urination and reduces edema. Promotes the discharge of pus, generates flesh and expels toxins. Generates body fluids. Relieves numbness and pain | Anti-inflammatory, antibacterial, anticancer, antioxidant, antiviral, cardiovascular effects, gastrointestinal effects, glucose-lowering, hepatoprotective, immunomodulatory, renoprotective, vasorelaxant, vulnerary. |
| Shengjiang | *Zingiber officinale* Rosc. | *Zingiberis Rhizoma Recens*;  Fresh ginger | Releases the exterior, induces perspiration and disperses cold. Warms the middle and stops vomiting. Warms the lungs and stops coughing. Reduces toxicity of other herbs and seafood. Adjusts the *yang wei,* normalises the flow of *qi* at the centre. | Analgesic, anthelmintic, antibacterial, anti-diabetic, anti-emetic, antifungal, anti-inflammatory, antioxidant, anti-platelet, hypotensive, cognitive enhancer, gastroprotective, immunomodulator, insecticidal, lipid-lowering, respiratory anti-microbial, anti-obesity. |
| Jili | *Tribulus terrestris* L. | *Tribuli Fructus*;  Caltrop fruit;  Puncture-vine fruit; Tribulus | Calms the liver and anchors *yang,* Dredges liver *qi* and disperses stagnation and clumping dispels wind-heat and brightens the eyes. Dispels wind and stops itching. Extinguishes liver wind. | Anthelmintic, anthelmintic, anticancer, antifungal, cardiovascular effects, hormonal effects, musculoskeletal effects, neurologic/central nervous system effects. |
| Juhua | *Chrysanthemum morifolium* Ramat. | *Chrysanthemi Flos*; Chrysanthemum flower | Disperses wind and clears heat. Calms the liver and clears the eyes. Calms liver and extinguishes *wind*. Promotes detoxification. Promotes movement of heart *qi* and blood and stimulates blood circulation. | Antidiabetic, antiviral, anti-inflammatory, antitumor, cardiovascular, antibacterial, anti-pyretic, hypolipidemic, hypotensive, ophthalmic effects, uric acid lowering effects. |
| Juemingzi | *Cassia obtusifolia* L.  *C. tora* L. | *Cassiae Semen*;  Cassia seed | Used to treat dizziness by anchoring liver *yang* and nourishing the liver. Moistens the intestines and unblocks the bowels. Reduces blood pressure and serum cholesterol. Expels wind-heat (Ali, Park, & Chang, 2021). | Anti-oxidant, anti-Parkinson’s, anti-inflammatory, neuroprotective, anti-cancer, anti-Alzheimer, hepatoprotective, anti-platelet, anti-microbial, anti-oxidant, anti-diabetic (Ali, Park, & Chang, 2021). |
| Luobumaye | *Apocynum venetum* L. | *Apocyni Veneti Folium*;  Dogbane leaf | Calms liver *yang*, clears liver fire and extinguishes wind. Clears heat and facilitates urination. Nourishes heart and calms the *shen*. Enhances health in the middle aged and elderly. | Hepatoprotective, anti-oxidant, apoptosis inducing, anti-hypertensive, anti-depressant, anxiolytic, anti-ageing, cardio-tonic (Xiang, Wu, Isah, Chen, & Zhang, 2023). |
| Mudanpi | *Paeonia suffruticosa* Andr. | *Moutain Cortex*;  Tree peony bark | Clears heat and cools (harmonises) the blood. Clears deficiency fire. Invigorates the blood and dispels blood stasis. Clears liver fire. Drains pus and reduces swelling. | In WHM same as *P. lactiflora.* |
| Niuxi | *Achyranthes bidentate* Bl. | *Achyranthis Bidentatae Radix* | Removes [blood stasis](https://www.sciencedirect.com/topics/pharmacology-toxicology-and-pharmaceutical-science/blood-stasis), dredges meridians, tonifies the liver and kidneys, strengthens muscles and bones, facilitates diuresis, and drains the blood (Fan, Wang, Zhang, Wu, & Chen, 2021). | Not commonly used is WHM. Exhibits anti-inflammatory, anti-diarrheal, anti-ageing, analgesic and anti-obesity (Fan, Wang, Zhang, Wu, & Chen, 2021). |
| Nvzhenzi | *Ligustrum lucidum* Ait. | *Ligustri Lucidi Fructus*;  Glossy privet fruit | Nourishes and tonifies liver and kidney *yin*. Clears deficiency heat. Improves vision. | Immunomodulatory, anti-tumour, immunostimulant, anti-inflammatory, anti-allergic, cardio-tonic, diuretic, sedative, lipid lowering, hypo-glycaemic, hepato-protective. |
| Renshen | *Panax ginseng* C. A. Mey. | *Ginseng Radix* et *Rhizoma*;  Ginseng root; Asian Ginseng root; Panax | Powerfully tonifies *yuan qi*. Tonifies spleen and stomach *qi*. Tonifies lung *qi*. Generates body fluids and stops thirst. Tonifies heart *qi* and calms the spirit. Treats impotence. Tonifies *qi* in deficiency patients with exterior conditions. | Alcohol clearance effects, anti-aging, anti-arthritis, anti-asthmatic, anti-cancer, anti-fatigue, anti-fungal, anti-inflammatory, anti-platelet, anti-stress, anti-viral, antibacterial, antidepressant, antidiabetic, antiemetic, antioxidant, antiulcer, blood pressure effects, cardiovascular protective, central nervous system effects, CYP450 effects, dermatologic effects, gastrointestinal effects, hepatoprotective, hypoglycemic, immune effects, lipid effects, memory effects, neuroprotective. |
| Roucongrong | *Cistanche deserticola* Y. C. Ma;  *C. tubulosa* (Schrenk) Wight | *Cistanches Herba*;  Desert living cistanche;  Desert ginseng | Tonifies the kidneys, strengthens *yang*, benefits *jing* and marrow and warms the womb. Moistens the intestines and facilitates passage of stool. | Anti-inflammatory, antioxidant, osteoporosis prevention, fatigue reduction, hepatoprotective, immunomodulatory, laxative, neuroprotective, increased testosterone. |
| Ruxiang | *Boswellia carterii* Birdw.;  *B. bhhaw-dajiana* Birdw. | *Olibanum*;  Frankincense;  Mastic; *Boswellia* | Invigorates the blood and promotes the movement of *qi*. Relaxes the sinews, activates the channels and alleviates pain. Reduces swelling and generates flesh. | Anti-Alzheimer, anti-arthritic, anti-asthma, anti-inflammatory, anticancer, immunomodulatory. |
| Shanyao | *Dioscorea opposita* Thunb. | *Dioscoreae Rhizoma*;  Chinese yam; Mountain yam rhizome | Tonifies the spleen, nourishes stomach *yin* and stops diarrhea. Tonifies lung *qi* and nourishes lung *yin*. Tonifies kidney *yin* and astringes *jing.* | Anti-osteoporotic, antidiabetic, gastrointestinal effects, hormonal effects, neurological effects. |
| Shanzhaye | *Crataegus pinnatifida* Bge. var. *major* N. E. Br.;  *C. pinnatifida* Bge. | *Crataegi Folium*; Crataegus; Hawthorn; Asian hawthorn; Mountain hawthorn | Reduces and guides out food stagnation and transforms accumulations. Transforms blood stasis, invigorates blood circulation and dissipates clumps. Stops diarrhea, treats hypertension, elevated serum cholesterol and coronary artery disease. | Antineoplastic, antioxidant, antiplatelet, cardiovascular effects, dermatologic effects. |
| Shengma | *Cimicifuga heracleifolia* Kom.;  *C. dahurica* (Turcz.) Maxim;  *C. foetida* L. | *Cimicifugae Rhizoma*;  Large trifoliolious bugbane rhizome;  Black cohosh | Releases the exterior and vent measles. Clears heat and relieves toxicity. Raises *yang* and lifts sunken *qi*. | Antihistamine, anti-inflammatory, antilipemic, antineoplastic, antioxidant, antiviral, osteoporosis prevention, CNS effects; phyto-estrogenic, gastrointestinal effects, hypotensive. |
| Shenqu | *Massa Medicata Fermentata* | Massa fermentata; Medicated leaven | Reduces food stagnation, promotes digestion and harmonizes the stomach. Moves *qi*. | Not used in WHM |
| Shudihuang | *Rehmannia glutinosa* Libosch. | *Rehmanniae Radix* Praeparata;  Prepared Rehmannia root | Nourishes the blood. Nourishes liver and kidney *yin.* Strongly enriches *yin* and relieves wasting and thirsting disorder. Nourishing *jing* and blood and fills the marrow. Arrests coughing and wheezing. | Anti-allergy, antidiabetic, antihypertensive, anti-inflammatory, antitumor, osteoporosis prevention, endocrine modulator, anti-ulcer (GIT), haematological properties, immunomodulator, neuroprotective, anti-depressant, anxiolytic. |
| Shouwuteng | *Polygoni Multiflori Caulis* | Polygonum vine/fleeceflower vine | Nourishes the heart *yin* and blood and calms the spirit. Unblocks channels and dispels wind-dampness. Alleviates itching (topical). Astringes sweet. | Anti-oxidant, antiatherosclerosis, anti-bacterial, anticolitis, anti-fibrotic, anti-inflammatory, anti-osteoporosis, anti-tumor, anti-viral, cholesterol lowering effects, oestrogenic effects, hypoglycemic, immunomodulator, laxative, hepatoprotective, neuroprotective, vasorelaxant, weight loss stimulation. |
| Shuinujiao | *Bubalus bubalis* Linnaeu | *Cornu bubali*;  Water buffalo horn | Clears heat from the *ying* and *xue* stages, relieves fire toxicity, cools the blood and stops bleeding. Clears heat and arrests tremors | Not used in WHM. |
| Shexiang | *Moschus berezovskii* Flerov;  *M. sifanicus* Przewalski;  *M. moschiferus* Linnaeus | *Moschus*;  Musk | Strongly opens the orifices and vessels, revives the spirit and unblocks closed disorders. Invigorates the blood, dissipates masses, reduces swellings, detoxifies, opens the channels and alleviates pain. Hastens delivery and facilitates the passage of stillborns (induces abortion) | Not used in WHM. |
| Suhexiang | *Liquidambar orientalis* Mill. | *Styrax* | Opens the orifices, penetrates turbidity, breaks up stagnation, clears filth and relieves pain. | Not used in WHM. |
| Suanzaoren | *Ziziphus jujuba* Mill. var. *spinosa* (Bunge) Hu ex H. F. Chou | *Ziziphi Spinosae Semen*;  Spine date seed | Nourishes heart *yin*, tonifies liver blood and calms the spirit. Astringent sweet and generates fluid. | Analgesic, anti-inflammatory, antidiabetic, antilipidemic, antimicrobial, antineoplastic, antioxidant, antiviral, cardiovascular, gastrointestinal effects, hepatoprotective, immune effects, neurological effects, weight effects, wound healing effects. |
| Taoren | *Prunus persica* (L.) Batsch;  *P. davidiana* (Carr.) Franch | *Persicae Semen*;  Peach seed | Breaks up blood stasis and invigorates blood circulation, moistens the intestines and unblocks the bowels, drains abscesses. | Not used in WHM. |
| Tianma | *Gastrodia elata* Bl. | *Gastrodiae Rhizoma*;  Dongguan | Rejuvenates the body, enhances health, and extends life without toxicity (Liu et al., 2018). Extinguishes *wind*, calms the liver and stops spasms and tremors. Extinguishes *wind*, subdues rising liver *yang* and alleviates pain. Disperses *wind*-damp. | Neuroprotective, anti-inflammatory, anti-oxidant, anti-epileptic, anti-convulsive, anti-psychotic, anxiolytic, anti-depressant, circulatory system modulating, cognition/memory improving effects (Liu et al., 2018). |
| Wuzhuyu | *Euodia rutaecarpa* (Juss.) Benth.;  *E. rutaecarpa* (Juss.) Benth. var. *officinalis* (Dode) *Huang*;  *E. rutaecarpa* (Juss.) Benth. var. *bodinieri* (Dode) Huang | *Euodiae Fructus*;  Medicinal euodia fruit | Warms the middle jiao, disperses cold, promotes the movement of *qi*, relieves stagnation in the liver channel and alleviates pain. Spreads the liver, descends rebellious *qi* and stops vomiting. Warms the spleen, stops diarrhoea and expels damp cold. Leads fire downwards. | Analgesic, antibiotic, anticancer, anti-inflammatory, cardiovascular effects, GIT effects, haematological effects, immune effects, neuroprotective, cognition/memory enhancement, anti-obesity/weight loss stimulation, uterine stimulant. |
| Xiakucao | *Prunella vulgaris* L. | *Prunellae Spica*;  Common selfheal fruit-spike | Clears liver and brightens the eyes. Clears hot phlegm and dissipates nodules. Treats hypertension. | Anticancer, antiviral, anti-inflammatory, antioxidant, antifibrotic, kidney protective, hepatoprotective. |
| Yimucao | *Leonurus japonicus* Houtt. | *Leonuri Herba*;  Chinese motherwort plant | Invigorates the blood, dispels stasis, regulates menstruation and reduces masses. Promotes urination and reduces swelling and oedema. Clears heat and resolves toxicity internally or topically. | Anticancer, anti-inflammatory, antimicrobial, antioxidant, antiplatelet, antiviral, diuretic, uterotonic. |
| Yuanzhi | *Polygala tenuifolia* Willd.;  *P. sibirica* L. | *Polygalae Radix*;  Thin-leaf milkwort root | Calms the spirit, sedates the heart and clears the channel joining the heart and kidney. Expels phlegm, opens orifices, clears the lungs and stops coughing. Reduces abscesses and dissipates swellings. | Anti-inflammatory, gastrointestinal irritant, respiratory reflex demulcent, neuroprotective. |
| Zexie | *Alisma orientalis* (Sam.) Juzep.;  *A. plantago-aquatica* Linn. | *Alismatis Rhizoma*;  Oriental water plantain rhizome | Promotes urination and leaches dampness. Settles ministerial *fire* in the kidneys by draining damp-heat from the lower *jiao*. Drains kidney *fire*. | Antiatherosclerosis, anti-oxidant, diuretic. |
| Zhenzhu | *Pteria martensii* (Dunker)  *Hyriopsis cumingii* (Lea)  *Cristaria plicata* (Leach) | *Margarita*;  Pearl shell layer | Sedates the heart and settles tremors and palpitations. Clears the liver and eliminates superficial visual obstructions. Promotes healing and generates flesh (topical). | Not commonly used in WHM. |
| Zhishi | *Citrus aurantium* L.  *C. sinensis* Osbeck | *Aurantii Fructus Immaturus*;  Immature orange fruit | Breaks up stagnant *qi*, reduces accumulation, descends *qi*, unblocks the bowels and removes stagnant food. Transforms phlegm, reduces distention and resolves hardenings. | Antiasthma, anti-inflammatory, antidiabetic, antilipidemic, antioxidant, cardiovascular effects, metabolic effects, prebiotic. |
| Zhizi | *Gardenia jasminoides* Ellis | *Gardeniae Fructus*;  Cape jasmine fruit | Clears heat, reduces fire and eliminates irritability in the san jiao. Clears heat and resolves dampness (drains damp-heat). Cools the blood and relieves toxicity (stops bleeding by astringing). Reduces swelling and invigorates the blood. | Anticarcinogenic, antidiabetic, anti-inflammatory, cognition enhancing, neuroprotective, anti-neuro-inflammatory, hepatoprotective, vulnerary. |
| Zhuru | *Bambusa tuldoides* Munro;  *Sinocalamus beecheyanus* (Munro) McClure var. *pubescens* P. F. Li;  *Phyllostachys nigra* (Lodd.) Munro  var. *henonis* (Mitf.) Stapf ex Rendle | *Bambusae Caulis In Taenias*;  Bamboo shavings | Clears heat and transforms hot phlegm. Clears heat and stops vomiting. Cools the blood and stops bleeding. Calms the fetus. | Anti-cancer effects, thyroid effects. |

Note: BPH-benign prostatic hyperplasia; CNS-central nervous system; GIT-Gastro-intestinal tract; WHM-Western herbal medicine; CHM-Chinese herbal medicine. All TCM actions from Penner, 2017 if not otherwise stated. All WHM from Therapeutic Research Centre, 2025 if not otherwise stated.

**Table S5** Adapted processing methods of Chinese herbal medicines table (Chinese Medicine Board of Australia, 2022).

| No. | Pin yin | English | Prefix/Suffix |
| --- | --- | --- | --- |
| 1 | Chao | Dry fried | Prefix |
| 2 | Cu | Processed with vinegar | Prefix |
| 3 | Fa | Processed with licorice and lime | Prefix |
| 4 | Duan | Calcinated | Prefix |
| 5 | Gan | Dried | Prefix |
| 6 | Jiang | Processed with ginger and alum | Prefix |
| 7 | Jio | Scorched | Prefix |
| 8 | Mi | Processed with honey | Prefix |
| 9 | Pao | Baked, blast-fried | Prefix |
| 10 | Tan | Charred | Suffix |
| 11 | Shu | Cooked | Prefix |
| 12 | Sheng | Unprocessed/fresh | Prefix |
| 14 | Zhi | Processed (using various methods) | Prefix |
| 15 | Zhi | Honey fried, fried with liquid adjuvant | Prefix |

**Reference**

Ali, M. Y., Park, S., & Chang, M. (2021). Phytochemistry, ethnopharmacological uses, biological activities, and therapeutic applications of *Cassia obtusifolia* L.: A comprehensive review. *Molecules*, *26*(20), 6252.

Bhattacharyya, N., Baugh, R. F., Orvidas, L., Barrs, D., Bronston, L. J., Cass, S., et al. (2008). Clinical practice guideline: Benign paroxysmal positional *Vertigo*. *Otolaryngology - Head and Neck Surgery*, *139*(5), S47–S81.

Chen, Y. J., Guo, Y. J., & Xu, D. G. ((2016).). Treatment for the *Vertigo* disease from the angle of the purge and clearing intestine for 20 cases. *Chinese Medicine Modern Distance Education of China*, *14*(9), 78–80.

Chinese Medical Education Association Vertigo Professional Committee. (2020).
Chinese expert consensus on the diagnosis and treatment of vascular dizziness/vertigo.
*Chinese Journal of Neuroimmunology and Neurology*, *27*(4), 253–260.

Chinese Medicine Board of Australia. (2022). Processing methods of Chinese herbal medicines. *Australian Health Practitioner Regulation Agency*.

Fan, S., Wang, Y., Zhang, Y., Wu, Y., & Chen, X. (2021). *Achyranthes bidentata* polysaccharide activates nuclear factor‑kappa B and promotes cytokine production in J774A.1 cells through TLR4/MyD88 signaling pathway. *Frontiers in Pharmacology*, *12*, Article 753599.

Hao, S. S., Wang, J., Xie, C. R., Wang, R., & Zhang, X. (2024). Randomized controlled trial of Qishen Zhixuan Formula in treating posterior circulation ischemia-induced isolated *Vertigo*. *Journal of Hunan University of Chinese Medicine*, *44*(11), 2056–2060.

He, X. R., Wang, J. H., Li, M. X., Hao, D. J., Yang, Y., Zhang, C. L., et al. (2014). *Eucommia ulmoides* Oliv.: Ethnopharmacology, phytochemistry and pharmacology of an important traditional Chinese medicine. *Journal of Ethnopharmacology*, *151*(1), 78–92.

Huang, Y. L., Wang, S. S., Liu, L., Peng, W., Wang, J. L., Song, Y., et al. (2019). Review of traditional uses, botany, chemistry, pharmacology, pharmacokinetics, and toxicology of *Radix* Cyathulae. *Chinese Medicine*, *14*(1), 17.

Kong, Q. H., Niu, Y. S., Feng, H., Yu, X. F., Wang, B. K., Liu, X., et al. (2024). *Ligusticum chuanxiong* Hort.: A review of its phytochemistry, pharmacology, and toxicology. *Journal of Pharmacy and Pharmacology*, *76*(11), 1404–1430.

Lei, H. B., & Zhang, H. ((2016).). Clinical efficacy of Banxia Baizhu Tianma decoction plus canalith repositioning procedure in treating benign paroxysmal positional *Vertigo* (BPPV) and their influence on blood lipid metabolism. *World Chinese Medicine*, *11*(10), 2039–2042.

Li, H., & Yang M. H. (2019), Clinical effect of self-made “Huatan Huoxue prescription” for the treatment of unexplained vertigo: A randomized, open-label, positive-drug control trial. *International Journal of Clinical and Experimental Medicine*, *12*(11): 12886–12895.

Liu, T. Z., Ren, W. Q., Geng, X. W., & Liu, C. G. (2026). *Uncaria rhynchophylla*: An ethnopharmacological review integrating traditional Chinese medicine uses with phytochemical and pharmacological evidence. *Frontiers in Pharmacology*, *17*, 1723499.

Liu, Y., Gao, J. L., Peng, M., Meng, H. Y., Ma, H. B., Cai, P. P., et al. (2018). A review on central nervous system effects of gastrodin. *Frontiers in Pharmacology*, *9*, 24.

National Administration of Traditional Chinese Medicine. (1994). Diagnostic and therapeutic efficacy criteria of traditional Chinese medicine diseases (p.23). Nanjing: Nanjing University Press.

Penner, J. (2017). American Dragon: Chinese herbs and traditional Chinese medicine. https://www.americandragon.com

Shorbagi, M., Fayek, N. M., Shao, P., & Farag, M. A. (2022). *Citrus reticulata* Blanco (the common mandarin) fruit: An updated review of its bioactive, extraction types, food quality, therapeutic merits, and bio-waste valorization practices to maximize its economic value. *Food Bioscience*, *47*, 101699.

Therapeutic Research Center. (2025). Natural medicines database. Therapeutic Research Center. https://naturalmedicines.therapeuticresearch.com

Wu, C. H., Liao, L. M., Yan, X. H., Li, M. D., Wu, S. Y., Wang, J. M., et al. (2013). Effects of Yangxue Qingnao Granules on chronic cerebral circulation insufficiency: A randomized, double-blind, double-dummy, controlled multicentre trial. *Psychogeriatrics*, *13*(1), 29–34.

Xiang, T., Wu, L. J., Isah, M. B., Chen, C., & Zhang, X. Y. (2023). *Apocynum venetum*, a medicinal, economical and ecological plant: A review update. *PeerJ*, *11*, e14966.

Xu, S. C. (2015). Clinical observation on combination of acupuncture with medicine treatment compared with intravenous drip of liver Yang syndrome *Vertigo* in 130 cases. *Guangming Journal of Chinese Medicine*, *30*(5), 968–970.

Zhang, L., Xu, Z. J., Jiang, T., Zhang, J. L., Huang, P. X., Tan, J. Q., et al. (2021). Efficacy and safety of Ejiao (asini corii *Colla*) in women with blood deficient symptoms: A randomized, double-blind, and placebo-controlled clinical trial. *Frontiers in Pharmacology*, *12*, 718154.

Zhang, Y. (2012). Clinical observation of integrated traditional Chinese and Western medicine treatment for migraine dizziness. *Chinese Journal of Emergency Medicine in Traditional Chinese Medicine*, *21*(4), 637–638.

Zhang, Y. J., Xu, L., Wang, X. P., Ma, J. J., Song, X. P., Lv, H., et al. (2023). The clinical efficacy of Shenxiang Suhe Pill administration in posterior circulation ischemic *Vertigo*: A randomized controlled trial. *Medicine*, *102*(51), e36604.

Zhang, Y. K., Chen, C. X., Huang, Y. C., Bian, Y. F., Liu, M. Y., Lü, H. H., et al. ((2020).). Clinical efficacy of Huzhangye capsule combined with manual reduction on benign paroxysmal positional *Vertigo*. *Academic Journal of Second Military Medical University*, *41*(11), 1298–1301.

Zhao, H., Feng, Y. L., Wang, M., Wang, J. J., Liu, T., & Yu, J. (2022). The *Angelica dahurica*: A review of traditional uses, phytochemistry and pharmacology. *Frontiers in Pharmacology*, *13*, 896637.
